# Genome-Wide Analysis of Promoters: Clustering by Alignment and Analysis of Regular Patterns

**DOI:** 10.1371/journal.pone.0085260

**Published:** 2014-01-22

**Authors:** Lucia Pettinato, Elisa Calistri, Francesca Di Patti, Roberto Livi, Stefano Luccioli

**Affiliations:** 1 Dipartimento di Fisica e Astronomia, Università degli Studi di Firenze, Sesto Fiorentino, Italy; 2 Istituto Nazionale di Fisica Nucleare, Sesto Fiorentino, Italy; 3 Centro Interdipartimentale per lo Studio delle Dinamiche Complesse, Sesto Fiorentino, Italy; 4 Dipartimento di Biologia, Università degli Studi di Firenze, Sesto Fiorentino, Italy; 5 Istituto dei Sistemi Complessi, Consiglio Nazionale delle Ricerche, Sesto Fiorentino, Italy; 6 Joint Italian-Israeli Laboratory on Integrative Network Neuroscience, Tel Aviv University, Ramat Aviv, Israel; University of Turin, Italy

## Abstract

In this paper we perform a genome-wide analysis of *H. sapiens* promoters. To this aim, we developed and combined two mathematical methods that allow us to (i) classify promoters into groups characterized by specific global structural features, and (ii) recover, in full generality, any regular sequence in the different classes of promoters. One of the main findings of this analysis is that *H. sapiens* promoters can be classified into three main groups. Two of them are distinguished by the prevalence of weak or strong nucleotides and are characterized by short compositionally biased sequences, while the most frequent regular sequences in the third group are strongly correlated with transposons. Taking advantage of the generality of these mathematical procedures, we have compared the promoter database of *H. sapiens* with those of other species. We have found that the above-mentioned features characterize also the evolutionary content appearing in mammalian promoters, at variance with ancestral species in the phylogenetic tree, that exhibit a definitely lower level of differentiation among promoters.

## Introduction

Non–coding regions of DNA contain important functional elements that mainly concern regulatory activities and changes in gene expression. Recently, such functionality has been defined as the participation in at least one reproducible biochemical event, for instance TF association, chromatin structure- or histone-modification [1]. Moreover, there is a widespread consensus in identifying the non-coding DNA as the major substrate for critical changes. They are expected to drive phenotypic modifications and differences between species or individuals, thus representing the basis for evolution as well as for disease-associated regulatory variants [2]–[5]. The variability of non-coding DNA appears to be correlated with organism complexity, thus supporting the conjecture that it is of primary importance for the genetic programming of complex eukaryotes [6], [7].

In the presence of this new challenging scenario for genomics, several research groups are nowadays devoting considerable efforts to the study of non–coding DNA regions. Traditional in silico approaches are based on comparative genomics, that relies upon evolutionary conservation as a basic property for identifying functional regions. For instance, pairwise or multiple sequence alignments have been used for predicting non-coding RNA transcripts or Transcription Factor (TF) binding sites [8]–[13]. By comparing genomic DNA from closely and distantly related species, functional elements may be recognized on the basis of their conservation. Comparative analyses can be applied also within a species to find paralogous regions deriving from duplication events within a genome [14] or even function-related patterns based on sequence similarities [15]. These sequence-based analyses, together with experimental techniques [16]–[18], have proved quite effective for predicting functional non-coding sequences and their biological implications [19]. On the other hand, as a consequence of the variability of regulatory regions, it is quite difficult to establish the accuracy of such methods in estimating the TF binding or the transcriptional output [20], [21]. In fact, it is well known that, at variance with coding sequences that are well conserved even across distantly related species, regulatory regions are relatively flexible, since most TFs tolerate considerable variations in target sequences [22]. The high turnover rate both in adjacent putatively non-functional DNA and in duplicated TF binding sites often disrupts sequence conservation and makes alignments impossible (e.g., see [23]–[25]. Moreover, transcriptional rewiring [26] may explain events of sequence similarity loss, but retention of similar function. Accordingly, in non-coding DNA, sequence homology may not necessarily correspond to functional homology.

For all these reasons the comparative approach among specific sequence elements in the non-coding regions of DNA is certainly useful, but insufficient to obtain an exhaustive description of DNA double helix functional properties. Many other approaches have been proposed to fill the gap. Among them we just mention the various techniques that run motif-finding algorithms on sets of sequences and incorporate the information of experimentally known TF binding sites in position-specific weight matrices [27]–[29], or rely on the study of the three–dimensional structure of DNA [30], [31] and on neural network optimization procedures [32], [33].

In this paper we focus our study on promoters, because they are known to play a crucial role in the expression and regulation of genes [1].

In two previous works [15], [34] evidence was found of a correlation between the properties of promoter sequences and the kind of genes they regulate. In particular, base composition analysis (BCA) and specific entropic indicators were employed for identifying structural similarities among different classes of promoters [35], [36]. Moreover, the region around the TSS was shown to exhibit a very distinctive structural profile, which seems to be actively maintained by non–neutral selective constraints. Such structural profile is primarily related to a non–random distribution of nucleotides along the promoter close to the TSS [15], [34]. Another relevant outcome of these analyses concerns the importance of the role played by the different chemo-physical properties of the weak and strong nucleotides, thus indicating a possible relation also with the mechanisms associated to the double helix opening and bendability [37]–[40].

In this paper we perform a genome-wide analysis of promoter sequences. In particular the analysis is focused on *H. sapiens* but a comparison with other species is also presented. To this aim, we developed and combined two mathematical methods that allow us to (i) classify promoters into groups characterized by specific structural features, and (ii) recover, in full generality, any regular sequence in the different classes of promoters. Our goal is to highlight the global properties of the promoters that, at variance with the DNA coding regions, appear as a combination of random assemblies of nucleotides, alternating with fairly regular sequences. We focus our attention on regular sequences because many of them have been shown to posses peculiar structural properties involved in regulatory functions [38], [39], [41], [41]–[44].

The first method makes use of a clustering algorithm, that groups promoters by exploiting an alignment procedure [45]–[47] that takes into account the whole sequence (see section *Spectral Clustering* 0 in Methods). The second method identifies regular sequences characterizing the different clusters. In this framework, the promoter is modelled as a chain of oscillators according to the Peyrard–Bishop model [48]–[50] (see section *Spectral method for identification of regular sequences* in Methods): from the analysis of the vibrational properties of the promoter chain it is possible to identify all the regular sequences.

In section *Clustering of promoters* we report the results of the clustering procedure and we show that *H. sapiens* promoters can be classified into four main groups featuring different structural properties. The next two sections (*Regular nucleotide sequences in promoters* and *Transposons and regular sequences*) are devoted to discussing the relevance of the different content of regular sequences in the four clusters detected. In particular, we show that the most frequent regular sequences in two of the four clusters are strongly correlated with transposons: this constitutes one of the main biologically relevant results reported in this manuscript. The results about the comparison among different species, extensively discussed in file Text S1, indicate that, even in mammals, the most frequent regular sequences are benchmarks for different species - a completely opposite situation with respect to the coding component of DNA that is highly conserved.

## Results and Discussion

### Clustering of promoters

The database of *H. sapiens* promoters used in this paper contains 32122 sequences associated to protein–coding genes (see section *Databases* in Methods). Each promoter is represented by the 1000 nucleotides upstream of the TSS of all annotated genes.

A first classification of the promoters of this database was proposed in [15]. It relied upon the heuristic criterion of subdividing the database into two classes determined by the presence of the TATA-box (see section *TATA–box* in Methods). This criterion was inspired by the conjecture that these two classes are usually related to different promoter regulatory activity, that is promoters containing a TATA-box are usually associated with tissue–specific genes, while TATA-less promoters are related to housekeeping genes [35]. The analysis of the average base composition and of suitable entropic indicators showed that promoters containing the TATA-box (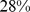 of the whole *H. sapiens* database) exhibit quite a different nucleotide composition (AT rich) with respect to the group of TATA–less promoters (CG rich) [35], [51]. This was a very interesting result, if one considers that the difference between these classes of promoters is not limited to the region close to the TSS, but it extends over the entire promoter. It was also pointed out that such differences are correlated with the presence of homogeneous sequences, whose composition characterizes the two groups of promoters [15].

In this paper we adopt a general clustering strategy of *H. sapiens* promoters that takes into account the global properties of the whole promoter instead of specific short regulatory motifs. The clustering procedure described in section *Spectral Clustering* of Methods is based on the spectral analysis of a similarity matrix: the entries of such matrix are obtained by an alignment algorithm that evaluates the similarity between promoters. The robustness of the method has been first verified by comparing two different alignment algorithms, namely Needleman–Wunsch [45] and Waterman-Smith [46]. We have found that, although the entries of the similarity matrix are quite different, both alignment algorithms yield essentially the same cluster organization. Accordingly, we have decided to report here only the result of the Needleman–Wunsch alignment algorithm, whose parameters have been fixed by a suitable optimization procedure (see section *Sequences alignment* in Methods). The main computational limitations of this clustering procedure stem from the alignment protocol and from the diagonalization of the similarity matrices. Therefore we have been able to consider similarity matrices of rank up to 2880, meaning that each run of the clustering algorithm can be applied to a sample of 2880 promoters. Taking advantage of the criterion employed in [15], each sample has been obtained by a random selection with equal probability of TATA and TATA–less promoters. This unbiased choice has been adopted to guarantee a comparison between numerically equivalent samples of sequences belonging to both promoter groups.

As shown in section *The Normalized Laplacian Matrix* in Methods, the eigenvalues of the Laplacian matrix, associated to the similarity matrix, highlight the presence of four clusters for *H. sapiens*.

**Figure 1:**
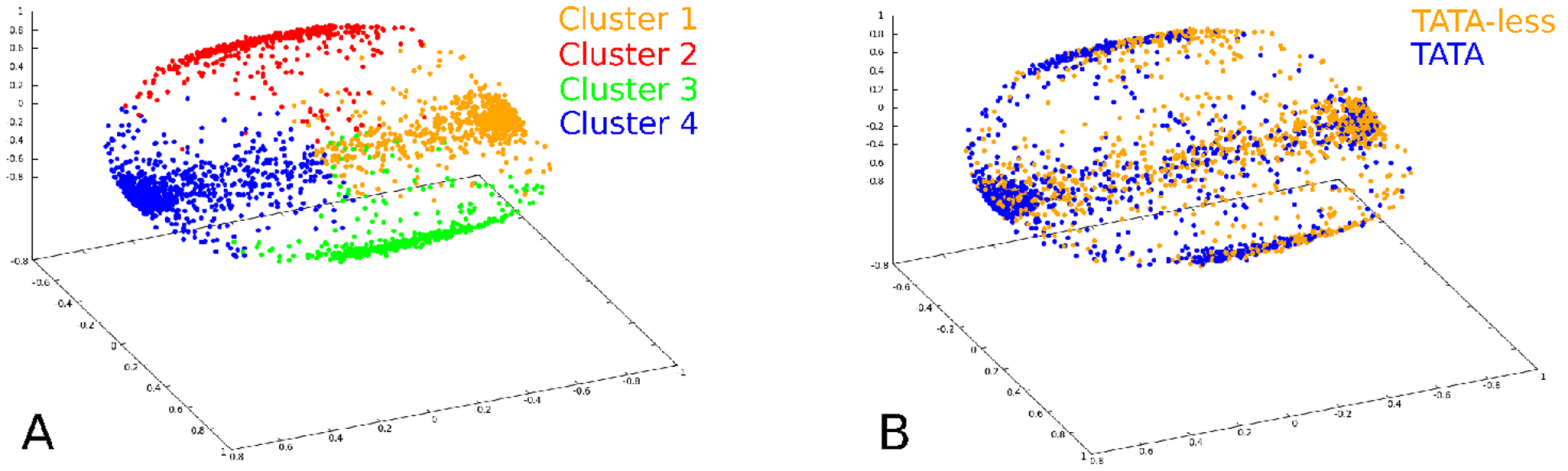
Distribution of points in the clustering space (see Methods) relative to the alignment of 2880 human promoters. Each point represents a promoter of the sample. **A**. The color code represents the four clusters. **B**. The color code represents the TATA (blue dots) and TATA–less (orange dots) classification.

We have also checked the robustness of the results by considering other sampling procedures. For instance, by choosing 28% of TATA and 72% of TATA-less promoters (according to their percentage in the database), or by a purely random sampling of promoters from the whole database, we still identify four clusters (apart from the number of promoters attributed to each cluster). In Fig. 1 we report the distribution of points in the clustering space. In this representation, as described in section *Clustering Algorithm* of Methods, each point represents a promoter: promoters with a high similarity score correspond to near points. Each of the 2880 promoters has been unambiguously associated to one of the four clusters with the procedure described in section *Clustering algorithm* of Methods. In panel A of Fig. 1 we make use of a four–color representation, where each color corresponds to a cluster, while in panel B we show, by a two-color representation, the partition into TATA and TATA–less promoters. The former (latter) are preferentially located on the left (right) side. Accordingly, we can conclude that our clustering algorithm yields a different and more refined classification of promoter sequences with respect to the mere partition of the sample into TATA and TATA–less promoters [15]. In fact, our clustering method takes into account global properties of promoters, while the one adopted in [15] relies upon a local criterion, i.e. the presence of the TATA–box in a specific promoter region.

**Figure 2:**
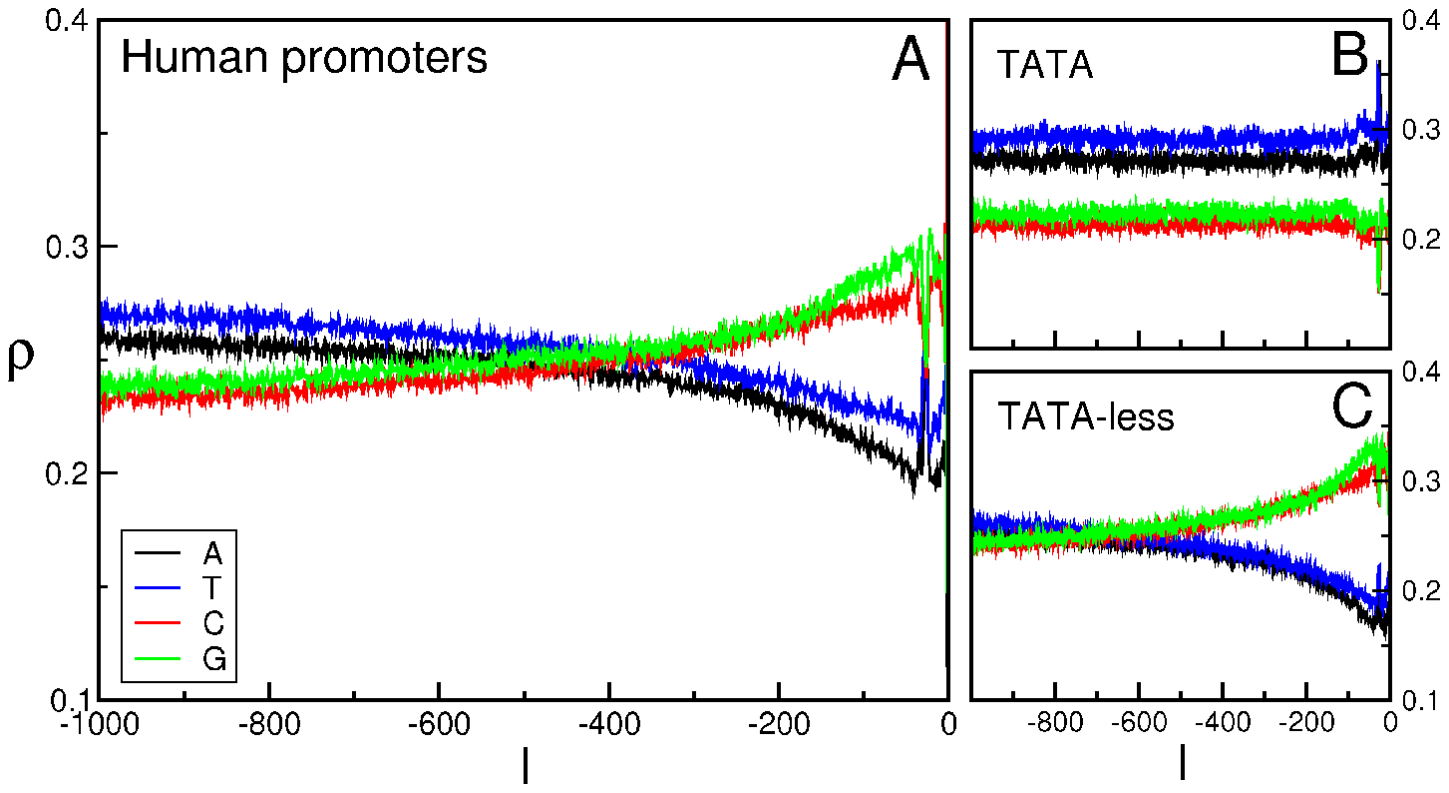
BCA of human promoters. BCA of the entire repertoire of human promoters (panel **A**) and of the two sets of TATA and TATA-less promoters (panels **B** and **C**). We report the frequency 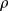 of each of the four nucleotides A (black), T (blue), C (red) and G (green) as a function of the position 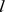 along the promoter (0 corresponds to the TSS). Figure from [15].

**Figure 3:**
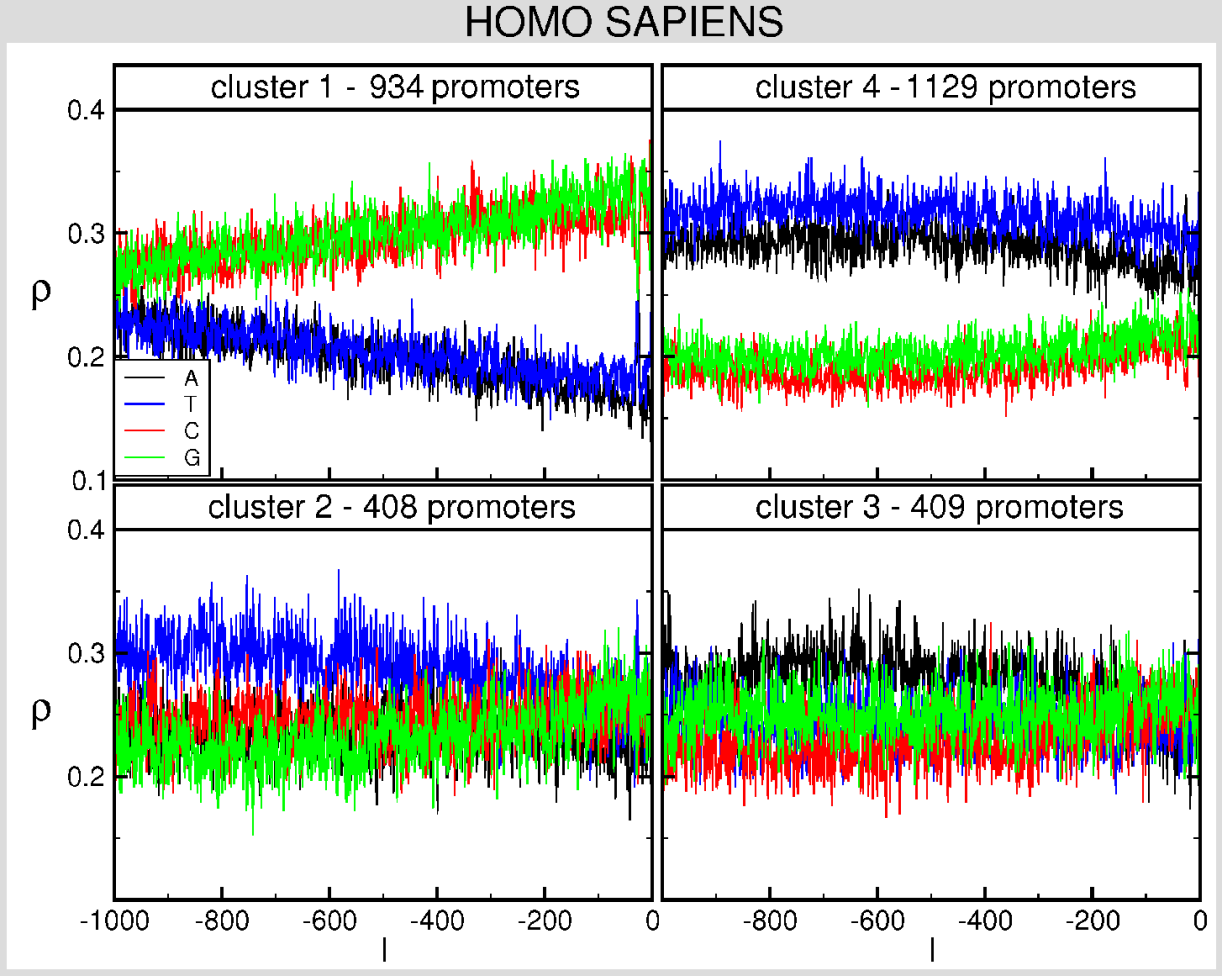
BCA of each of the clusters obtained with the clustering algorithm for *H. sapiens*. We report the frequency 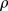 of each of the four nucleotides A (black), T (blue), C (red) and G (green) as a function of the position 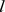 along the promoter (0 corresponds to the TSS).

**Figure 4:**
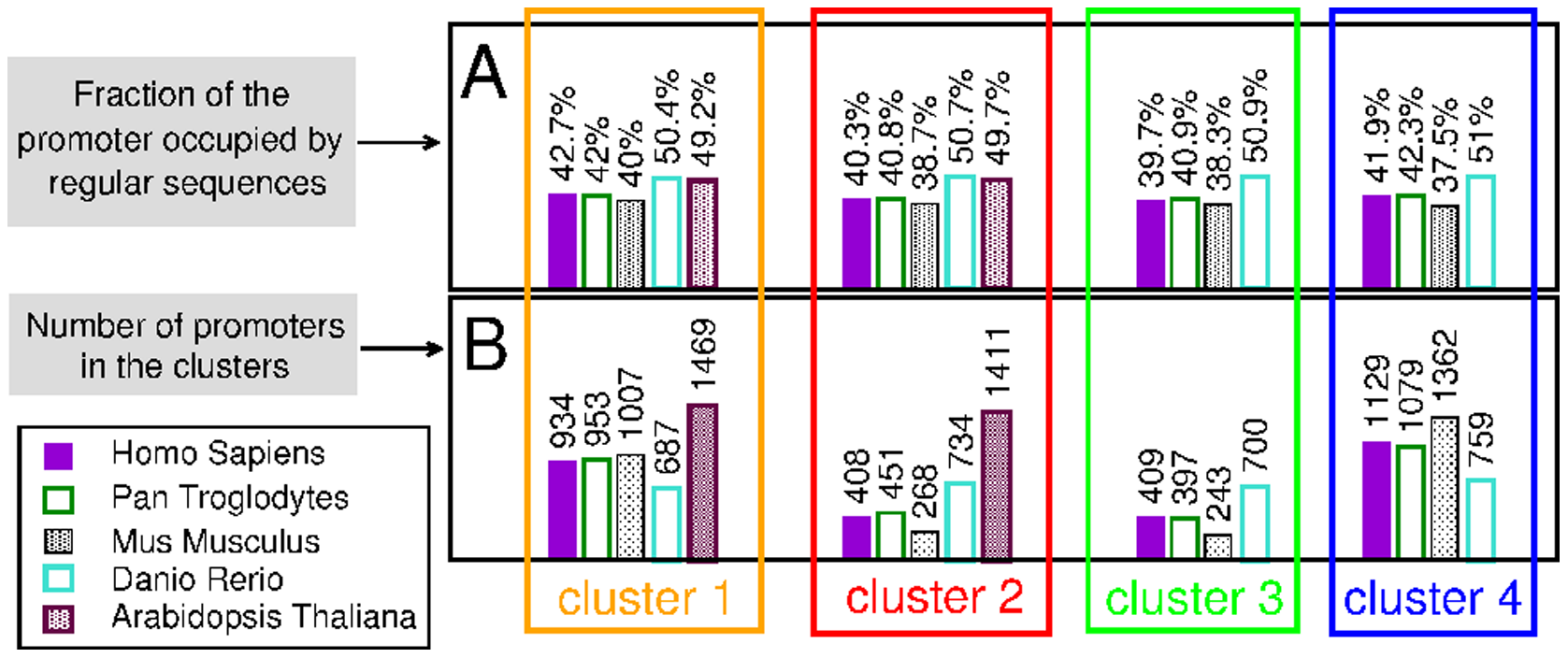
Occurrence of regular sequences in the clusters of promoters of different species. **A.** Average fraction of the promoter occupied by regular sequences. **B**. Number of promoters within the clusters.

Such a difference also emerges from the comparison of the BCA for the two families of TATA and TATA-less promoters of the whole database (see Fig. 2) with the one of promoters in the four clusters (see Fig. 3). The latter exhibit two clusters dominated by CG and AT nucleotides, denoted as cluster 1 (C1) and cluster 4 (C4), respectively; the other clusters, 2 (C2) and 3 (C3), on the contrary, are characterized by a more uniform distribution of nucleotides. The clusters (that correspond to those of panel A of Fig. 1) contain 934 (C1), 408 (C2), 409 (C3) and 1129 (C4) promoters (see also Fig. 4). We observe a different content of TATA promoters in each clusters: in C1 the percentage is about 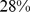, in C4 is 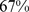 while in C2 and C3 it is 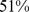.

It is known from the literature that the region around the TSS of animal promoters is typically CG enriched [52], [53]. On the other hand, the result of our clustering procedure indicates that a strong CG bias is present all along the extension of a specific subset of promoters, i.e. those contained in C1. Although a commonly accepted explanation of the CG enrichment in mammalian promoters is the presence of the so-called CpG islands, in a previous work [15] it has been shown that all the strong dinucleotide combinations increase with the same rate towards the TSS in mammalian promoters. The same scenario is recovered here for the promoters in C1 (see Fig. S11). For more details, see section *CpG dinucleotide analysis* in file Text S1.

The same partition into four clusters has been obtained also for *P. troglodytes* and *M. musculus* (see Fig. S1). This suggests that, at least for mammals, there is a general organization of promoters into structurally similar clusters.

This clustering method, that takes into account the entire promoter, has been applied also to species different from mammals. For instance, we have studied *D. rerio* and *A. thaliana*, but in this case we do not observe any indication of a clustering. As shown in section *Clustering and BCA of other species* in file Text S1, a clustering for these species can be recovered by limiting the alignment algorithm to a shorter and more specialized region of the promoter, i.e. the first 100 nucleotides upstream the TSS. This seems to suggest that regions much further than 100 nucleotides from the TSS can be considered intergenic regions, that do not correspond to any specific function. This conjecture is also confirmed by other studies of the functional regions of the genome in different species [15], [22], [34].

Altogether, the clustering analysis indicates that promoters in mammals exhibit common features, that depend on global structural properties. Conversely, in other species the clustering strategy is effective only when limited to relatively small regions (typically 100 nucleotides) close to the TSS.

Now, the main question concerns the identification of the structural features characterizing the different clusters.

### Regular nucleotide sequences in promoters

The complex structure of nucleotide sequences in promoters is due to the alternation of regular and disordered regions. As discussed in Methods (see section *Spectral method for identification of regular sequences*), these regions can be completely identified by computing the eigenvalues and the eigenvectors of the Hessian Matrix derived from the harmonic approximation of a simple double-strand DNA model [48]–[50]. The parameters of the model have been chosen according to phenomenological information. One major limitation of this simple model is that it can distinguish only between weak and strong nucleotides. It could be argued that this binary representation introduces a strong bias, because a regular sequence in a weak (W) and strong (S) binary code is not necessarily regular in the natural (A, T, C, G) quaternary code. On purely heuristic grounds, we can say that in most of the promoters many “regular” sequences in the binary code are still “regular” in the quaternary code. Moreover, as testified by the results discussed hereafter, we have checked that a good deal of the regular sequences in the binary code (that may appear less regular in the quaternary code) still play a relevant role in characterizing structural features of the different clusters. We want to recall that the use of the (W, S) binary code has revealed effective also for analyzing promoter sequences by entropic indicators [15].

We have found that regular sequences are distributed all along the promoters and cover a relevant portion of them: on average, about 40% of the promoter length in *H. sapiens*, *P. troglodytes* and *M. musculus*, while they reach 50% in *D. rerio* and *A. thaliana* (see Fig. 4).

In this section, we focus on the investigation of the properties of the regular sequences in the four clusters of *H. sapiens*. Although they have been identified in the (W,S) binary code, it is worth representing them in the natural quaternary code. Given the huge number of regular sequences in each cluster (see sec. *Global statistics on regular sequences in H. sapiens* in file Text S1), we decided to focus our analysis only on the 15 most frequent regular sequences, conjecturing that their overrepresentation is related to their importance. Anyway, we do not claim that they are the only interesting ones.

**Figure 5:**
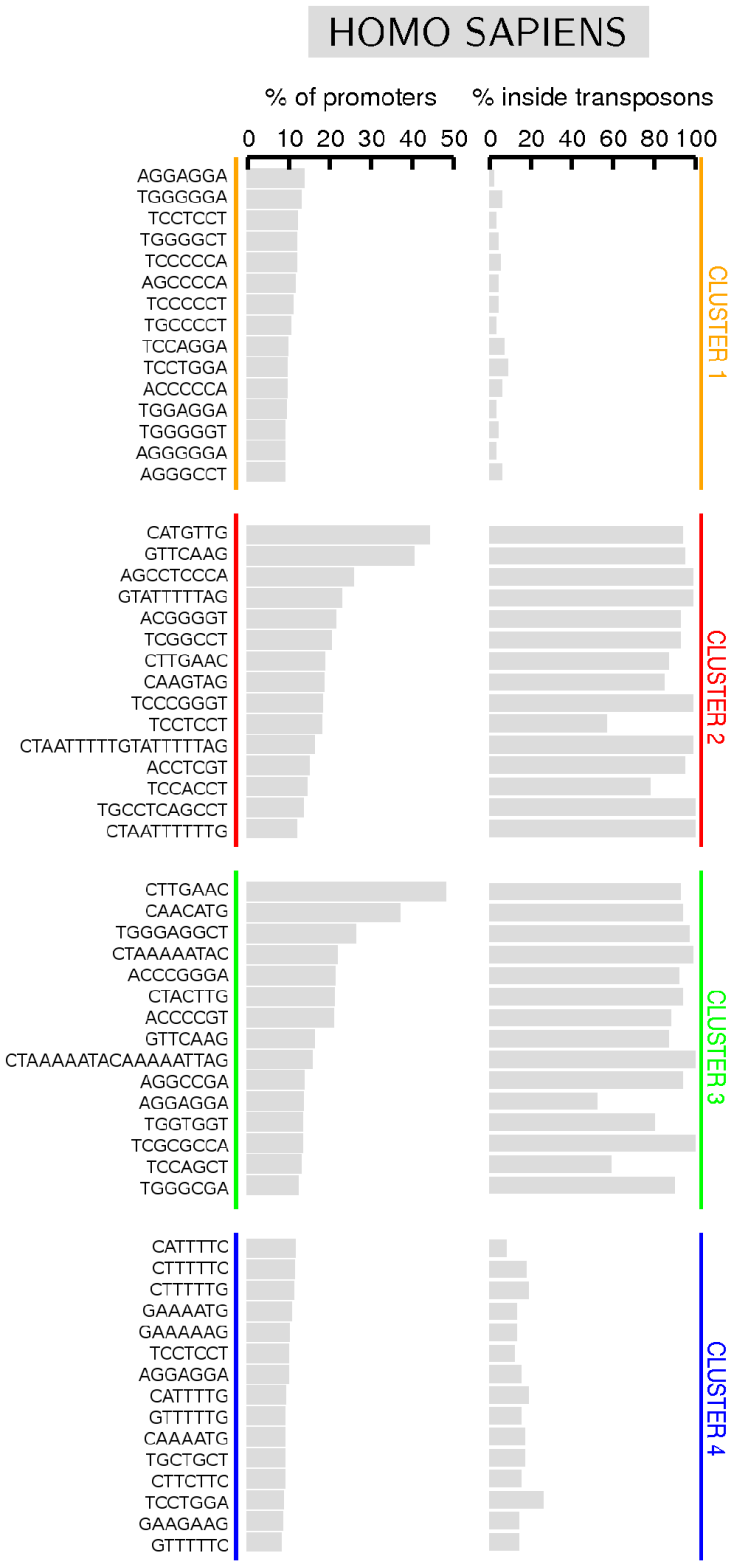
The most frequent regular sequences found in the clusters of *H. sapiens*. We report the percentage of promoters of the cluster in which the sequence appears at least once (left column), and the percentage of times the sequence is found inside a transposon (right column): it is calculated dividing the number of times it appears in a transposon by the total number of times it appears in the cluster.

The most frequent regular sequences found in C1 and C4 (see Fig. 5) extend over 7 nucleotides, i.e. the minimum length of a regular sequence detected by the algorithm (see section *Determination of regular sequences* in Methods). These short sequences exhibit a prevalence of S-nucleotides in C1 and of W-nucleotides in C4, consistently with the results obtained from the BCA (see Fig. 3). Their structure as well as their frequency in C1 and C4 are essentially similar. In most cases they are composed of a homogeneous sequence of five nucleotides flanked by two identical nucleotides of different nature in the (W,S) binary code, namely TCCCCCT, ACCCCCA, TGGGGGT, AGGGGGA, CTTTTTC, GAAAAAG, GTTTTTG. A first interesting quantitative feature is that each of these sequences appears in approximately 10% of the promoters of the cluster (see Fig. 5). We have also counted how many times each sequence is contained in these host promoters. The large majority contain the regular sequence just once, while only a small fraction of them contains it at most twice. In fact, the average number of each of these regular sequences in host promoters amounts to approximately 1.1: this indicates that each sequence is mostly spread across different promoters.

Also sequence AGGAGGA (as well as its complementary TCCTCCT) appears among the most frequent ones in all clusters. This sequence is fundamental in Prokaryotes, since it corresponds to a consensus sequence for the ribosome-binding site [54]. Its structural properties have been investigated [55], [56] together with its presence in promoters, where it has been found to interact with a stage-specific factor during the late stages of erythropoiesis [57].

One could wonder if overexpressed regular sequences in C1 and C4 are correlated with any biologically relevant function. For instance, taking inspiration from the literature, they could be associated with structural properties of the double helix [41], [43], [58], with the binding of basal transcription factors and RNA polymerase to DNA [21], [44], or to the possibility that homogeneous tracts could play the role of hotspots for mutations [42], [59]. On the same ground, one cannot exclude that they could interact with specific TF [21], [60]: we have checked this possibility with various tools and databases (i.e., [61]–[63]), but we have not found unambiguous outcomes corresponding to these motifs. Anyway, a verification of such conjectures is worthwhile, but goes beyond the aims of this manuscript and will be considered elsewhere. On the other hand, we have selected these sequences on the basis of their regularity and frequency, so that they are not necessarily associated with the specificity of regulatory signal typical of a TF binding site. In their turn, TF binding sites are variously dislocated along the genome (in enhancers, introns, etc.) and they are niether necessarily overexpressed nor regular, as they need a high information content for the specificity of their signal [20], [21].

Anyway, more relevant features differentiate C1 and C4 from C2 and C3, whose regular sequences typically exhibit a different structure. First of all, in C2 and C3 there are long regular sequences, up to 19 nucleotides (i.e. CTAATTTTTGTATTTTTAG and CTAAAAATACAAAAATTAG), among the most frequent ones. Moreover, the most frequent regular sequences appear in about 48% of promoters, at variance with C1 and C4, where they cover at most 14% of the promoters of the cluster. Last but not least, almost all regular sequences found in C2 have a companion sequence in C3 that corresponds to its reverse complement. As we are going to discuss in the following section, this observation indicates a relation of the most frequent regular sequences in C2 and C3 with transposons. This is by far the most interesting and distinctive feature of regular sequences in C2 and C3.

We want to conclude this section by adding two remarks.

Our analysis indicates that the clustering algorithm is able to detect specific similarities among promoters. In C1 and C4 similarities seem to stem just from the prevalence of S or W nucleotides, respectively, while in C2 and C3 they are mostly associated to the presence of specific regular sequences.

With regard to the comparison with other species, we want to point out that *P. troglodytes* exhibits the same most frequent regular sequences (including the 19–nucleotide one) found for *H. sapiens*. However, *M. musculus* exhibits rather different features (see Fig. S3). In *D. rerio* and *A. thaliana* the search for regular sequences has been performed in all the 1000 nucleotides of each promoter, even if the clusters differentiate only in the 100 nucleotides upstream the TSS. We have found that, at variance with mammals, the most frequent regular sequences are essentially the same in all the clusters (see Fig. S4). This is not completely unexpected, because of the low level of differentiation between promoters outside a small region near the TSS.

### Transposons and regular sequences

In order to identify correlations of regular sequences with specific elements in promoters, we have focused our attention on transposons, that are conjectured to be associated with promoter evolution, while playing a role in gene regulation and expression [64]–[66]. In fact, the observation of the reverse complementarity of regular sequences in C2 and C3 corresponds to a typical feature of transposons, that can indifferently intrude on both of the DNA strands. It is worth to recall here that the promoters in the database of *H. sapiens* belong to a specific strand (see section *Databases* in Methods).

**Figure 6:**
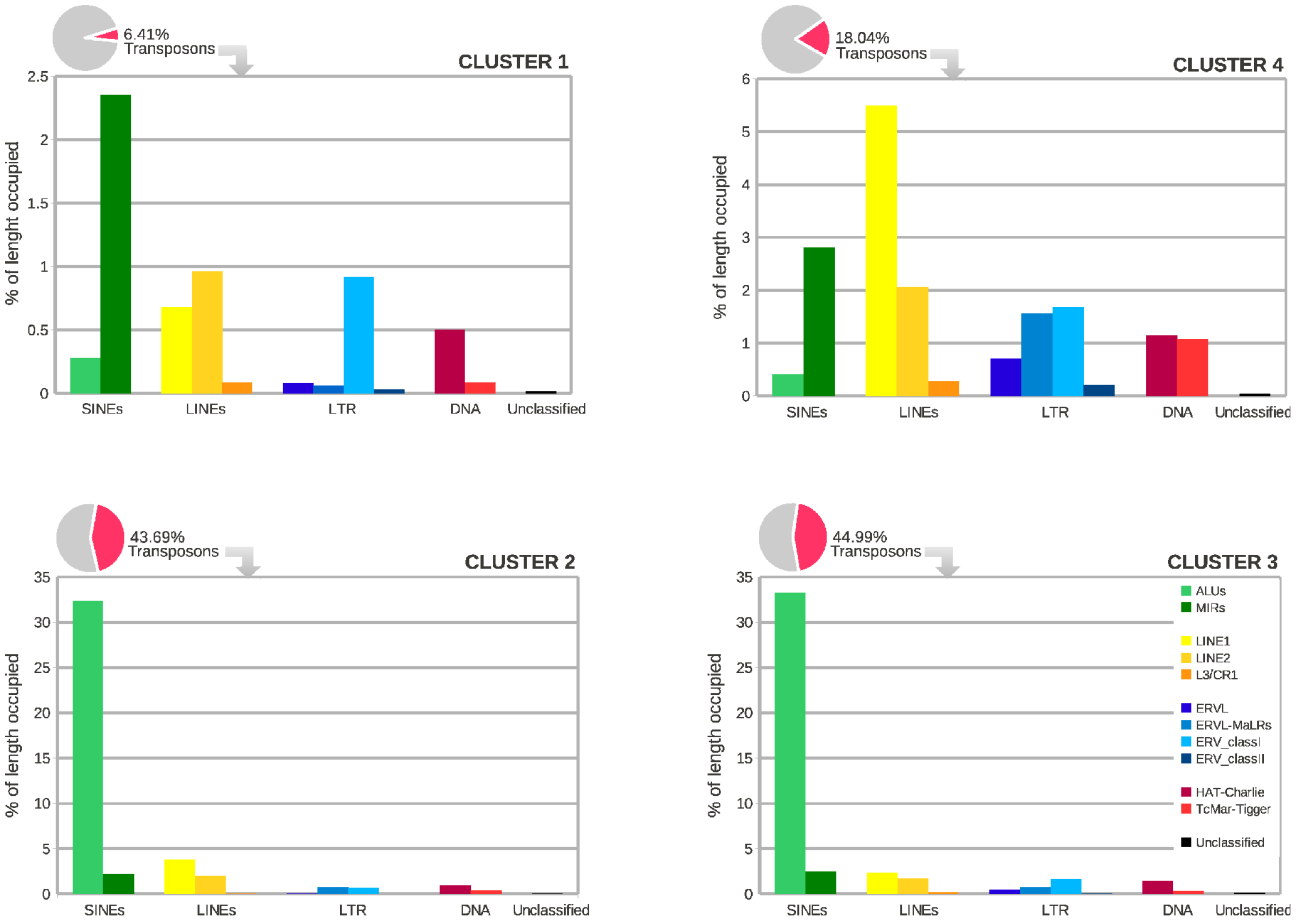
Distribution of the different families of transposons in the four clusters of *H. sapiens*. We report the total percentage of nucleotides in the cluster covered by transposons (pie chart) and the percentage of nucleotides covered by each family of transposons (histogram). Note the different scales in the histograms.

First of all we have identified (via the RepeatMasker software [67]) all transposons present in the promoters distributed in the four clusters of *H. sapiens*. We have found that C2 and C3 contain a large number of transposons, with a majority of Alu ones. On the contrary, C1 and C4 contain a smaller number of transposons, where Alu are quite rare (see Fig. 6).

The overabundance of Alu elements found in C2 and C3 could be read as a straightforward consequence of the fact that the Alu family is the most frequent dispersed repeat of the human genome: over one million copies of repeat elements, with a non–uniform distribution [65]. Our results have the merit of identifying the biases in their distributions among the different clusters of promoters.

A similar scenario is observed also for *P. troglodytes* and *M. musculus*, while in *D. rerio* and *A. thaliana* the content of transposons is approximately the same in all clusters (see Figs. S5, S6, S7, S8).

In order to disclose the conjectured relation between the most expressed regular sequences in C2 and C3 and transposons, we performed the following analysis. First, we computed the percentage of times each sequence belongs to a transposon (reported in the right column of Fig. 5). Then, we compared this result with the percentage of the cluster covered by transposons, which represents an estimation of the percentage we would expect if the sequence were equally distributed inside and outside the transposons. We have found that in C2 and C3 all the most expressed regular sequences appear in transposons with frequency much higher than the fraction of the cluster covered by transposons (that amounts to 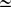
44–45%). Therefore, such sequences are much more likely to be located inside than outside a transposon: in some cases the probability is actually close to 1. In particular, the sequences with the highest probabilities (e.g. CTAATTTTTGTATTTTTAG) belong to the aforementioned Alu family. This is a strong indication that Alus are responsible of the enrichment of C2 and C3 with these specific sequences. On the other hand, the same analysis performed on clusters C1 and C4 shows that the most frequent regular sequences appear essentially equally distributed inside or outside the transposons. Altogether, we have obtained evidence that such distinctive features are strongly related to the discrimination of the different clusters in *H. sapiens*. Moreover, according to this observation, C2 and C3 should be considered as a unique cluster: as already mentioned, their apparently different features are the mere consequence of the insertion of transposons in different promoter strands, that yields the reverse complementarity characterizing regular sequences in these clusters.

In section *Transposons* of file Text S1 we have reported also the results obtained via the RepeatMasker software [67] for the other species considered in this paper, namely *P. troglodytes*, *M. musculus*, *D. rerio* and *A. thaliana*. For the first two species we observe very similar features with *H. sapiens*: in Fig. S3 we show that the correlation between the most-expressed regular sequences in C2 and C3 and transposons is preserved. In accordance with the known divergence of transposable elements between primates and mice [25], [68], the regular sequences in C2 and C3 of *M. musculus* are in most cases different from those of *H. sapiens* and *P. troglodytes*.

In the two other species transposons are equally distributed in all clusters. There is still a correlation between some regular sequences and transposons in *D. rerio*, while such a correlation is absent in *A. thaliana*.

## Conclusions

In this manuscript we performed a genome-wide analysis of *H. sapiens* promoters by exploiting a fully general mathematical procedure based on the combination of two spectral methods. The first one amounts to a clustering algorithm that allows us to classify promoters according to global similarities. The second spectral method is capable of detecting any regular sequence in each promoter, without imposing any preliminary constraint. The clustering analysis showed that *H. sapiens* promoters can be pooled into four main groups. Two of the clusters are distinguished by the prevalence of weak or strong nucleotides and are characterized by short compositionally biased sequences. In the two remaining clusters regular regions are found to be correlated with transposons, that are known to play a major role in favoring evolutionary changes in cis-regulatory regions, as conjectured by some authors [25], [64], [65], [68]. A posteriori, we are therefore led to conclude that these two clusters actually represent a single one.

In summary, the main biologically relevant findings consist in the following: (i) promoters can be classified according to common global properties of the whole sequence and not on the basis of the presence of specific patterns in specific positions (as for example in the usual TATA/TATA-less classification or other specific short regulatory motifs); (ii) promoters with the highest content of transposons group together in C2 and C3; (iii) the most expressed regular sequences of these clusters are essentially located inside transposons; (iv) conversely, in clusters C1 and C4 (where strong and weak nucleotides are respectively dominant) the most expressed regular sequences appear equally distributed along the promoters without any specific relation with transposons. Moreover, the generality of the unbiased methods, presented in this manuscript, allowed us to extend them to the investigation of promoter databases of other species. In file Text S1 we showed that the comparison of *H. sapiens* with other mammalian species points out that such species seem to be generally characterized by the presence of the same cluster organization. On the other hand, while the promoter structural properties of *H. sapiens* and *P. troglodytes* are almost identical, we find that *M. musculus* exhibits some differentiation in the most frequent regular sequences as well as in the correlation with transposons. An even more pronounced differentiation with respect to mammalian species is found in the promoters of a fish, *D. rerio*, and of a plant *A. thaliana*. At variance with mammalian promoters, where the information content spreads all over the promoter length, we have found that the clustering of promoters in these latter species is associated with a relatively short region (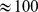 nucleotides) close to the TSS. Such a sharp differentiation of promoters structure in different species indicates that these DNA components are suitable candidates also for investigating the effects of evolutionary selection on DNA.

All the above mentioned results pave the way to new investigations. For example, some of the found regular sequences, because of their close–to–homogeneous composition, can be associated with known functional patterns: for instance, just to cite some examples, it is well known the effect of poly(A) sequences on the bendability of the double helix and the wrapping around nucleosomes [43], [58]; moreover in [41] authors claim that poly(dA∶dT) and poly(dC∶dG) tracts have a higher propensity for nonspecific TF-DNA binding, speeding up the stochastic search process for specific TF binding sites.

On the other hand, one cannot exclude that other regular sequences could also be eventually found to be associated with yet unexplored functional properties. More generally, one can guess that the overall ensemble of regular sequences constitute a sort of *substrate* with peculiar conformations, in which more irregular disordered sequences, endowed with a higher information content, are dispersed and play their role in specifying the regulatory signal [20], [44]. Accordingly, one could further guess that irregular and regular sequences in promoters may undergo different evolutionary processes: the first ones need sequence conservation, while the others may tolerate sequence variability. In the latter case conservation may involve certain conformational properties conferred for instance by sequence composition and correlations, periodicity, the length of regularity etc. We want to conclude by stressing again that the unbiased methods presented in this paper can be applied independently from conservation hypothesis or motif knowledge.

## Methods

### Databases

The promoters of *H. sapiens*, *M. musculus* and *D. rerio* have been downloaded from DBTSS (Version 6.0), a database of TSSs, obtained from a collection of experimentally-determined 5′-end sequences of full-length cDNAs [69]. *P. troglodytes* promoters have been downloaded from ECRbase, a database which provides a comprehensive collection of promoters generated by using expressed sequence tag (EST) and mRNA data [70]. The promoters of *A. thaliana* have been downloaded from a database where annotation of genes is largely based on sequenced cDNAs and ESTs alignments with the genome, that is TAIR (The Arabidopsis Information Resource) web site [71] (released in March 2008).

### TATA–box

Following Yang et al. [72], the TATA-box consensus sequence has been searched from position 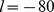 to 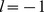 in the top strand of each promoter by an exact–match search. It corresponds to the degenerate sequence HWHWWWWR (coded according to IUPAC nomenclature), which identifies 576 sequences (in the nucleotide quaternary code). In order to fit the structural definition of the interaction with the TATA–binding protein, 44 specific strings have been excluded, so that the actually employed sequences reduce to 532 elements. Each promoter is called TATA if a TATA box consensus sequence is found at least once, otherwise it is called TATA-less. We have searched all the same degenerate boxes in the sets of promoter sequences of all the investigated species.

### Spectral clustering

The aim of the procedure described in this section is to divide the collected promoters into groups depending on the similarity between the sequences. The method is structured into three main steps: the first one consists in aligning each sequence with all the others (pairwise alignment) so as to obtain a matrix of similarity scores. Then, the analysis of the spectral properties of the Laplacian matrix calculated from the similarity matrix enables one to determine the appropriate number of groups for the clustering procedure. The last step, based on the k–means algorithm, associates each sequence to one of the clusters.

#### Sequences alignment

The basic idea of a sequence alignment is to identify regions of similarity that may be related with functional or structural properties as well as evolutionary relationships. Clearly, any alignment procedure cannot be based on a perfect match between sequences, but it has to take into account important biological features such as mutations and insertions or deletions occurred during the evolution. For this reason, the standard approach to this problem is to implement computational methods that make use of a substitution matrix to assign positive and negative scores to nucleotide matches or mismatches, and a gap penalty for matching a nucleotide in one sequence to a gap in the other one. These algorithms, in general, fall into two categories: global and local techniques. A global algorithm spans the entire length of the sequence, while a local alignment focuses on identifying regions of similarity within long sequences that are often widely different overall. In this paper we have made use of the two most popular alignment methods, the Needleman–Wunsch global algorithm [45] and the Smith–Waterman local algorithm [46] implemented in the EMBOSS package version 6.3.1 [47].

A key aspect of the procedure, which may give rise to a marked difference in the best match score calculated by the two algorithms, is the choice of the penalty value to be assigned to the introduction of a new gap in the alignment (GAPOPEN) and the value for each consecutive gap (GAPEXTEND); the scoring matrix for the nucleotide substitution has been taken equal to the standard EDNAfull matrix for both methods. Unfortunately there's no way to set a priori the optimal choice of parameters and thus the best option is to tune the values depending on the results obtained. Regarding our work, the trials we performed suggest to use a high GAPOPEN value (typically set equal to 20) and a low GAPEXTEND penalty (0.5 or 1) in order not to penalize long gap sequences. This setting favors the scores of very similar sequences yielding an easier detection of the correct number of clusters (see section *The normalized Laplacian matrix*). Moreover, in the EMBOSS code, gaps inserted at the beginning or at the end of the sequence have no penalty. In this way, we do not observe a significant difference between the two algorithms, and the outcome of aligning 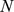 promoters gives the same similarity matrix 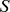 in both cases.

#### The normalized Laplacian matrix

A convenient way to represent the 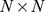 entries 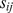 of the symmetric similarity matrix 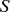, is to introduce a network whose nodes coincide with the sequences, while the entry 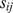 represents the weighted link between sequence 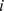 and 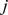. For the purpose of our work, however, dealing with a full connected network is not the best approach. The risk is that the noise induced by the fact that even the alignment of two random sequences gives a positive score, may hide the real common features among promoters, making the clustering procedure unfruitful. For this reason, it is of paramount importance to substitute 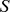 with a weighted adjacency matrix 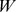, for which two nodes are connected only if their alignment score is larger that a certain threshold 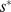, namely 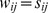 if 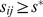 and 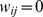 otherwise. To estimate 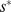, we have associated to each set of 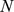 analyzed promoters, the corresponding 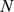 reshuffled sequences, namely the sequences obtained random rearranging the nucleotides of each promoters. Then we have performed the alignment, and calculated 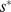 as the arithmetic mean of 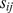. To check the correctness of 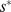, we have monitored 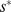 as a function of 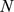 and we have observed the convergence of 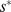 to a constant value for 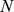 approaching the values used in our simulations (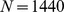, 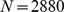; the choice 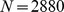 is due to the constraints on both the computational time and the size of the matrix to be stored). Finally, in order to manage a set of more homogeneous data, we have operated the normalization 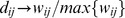.

**Figure 7:**
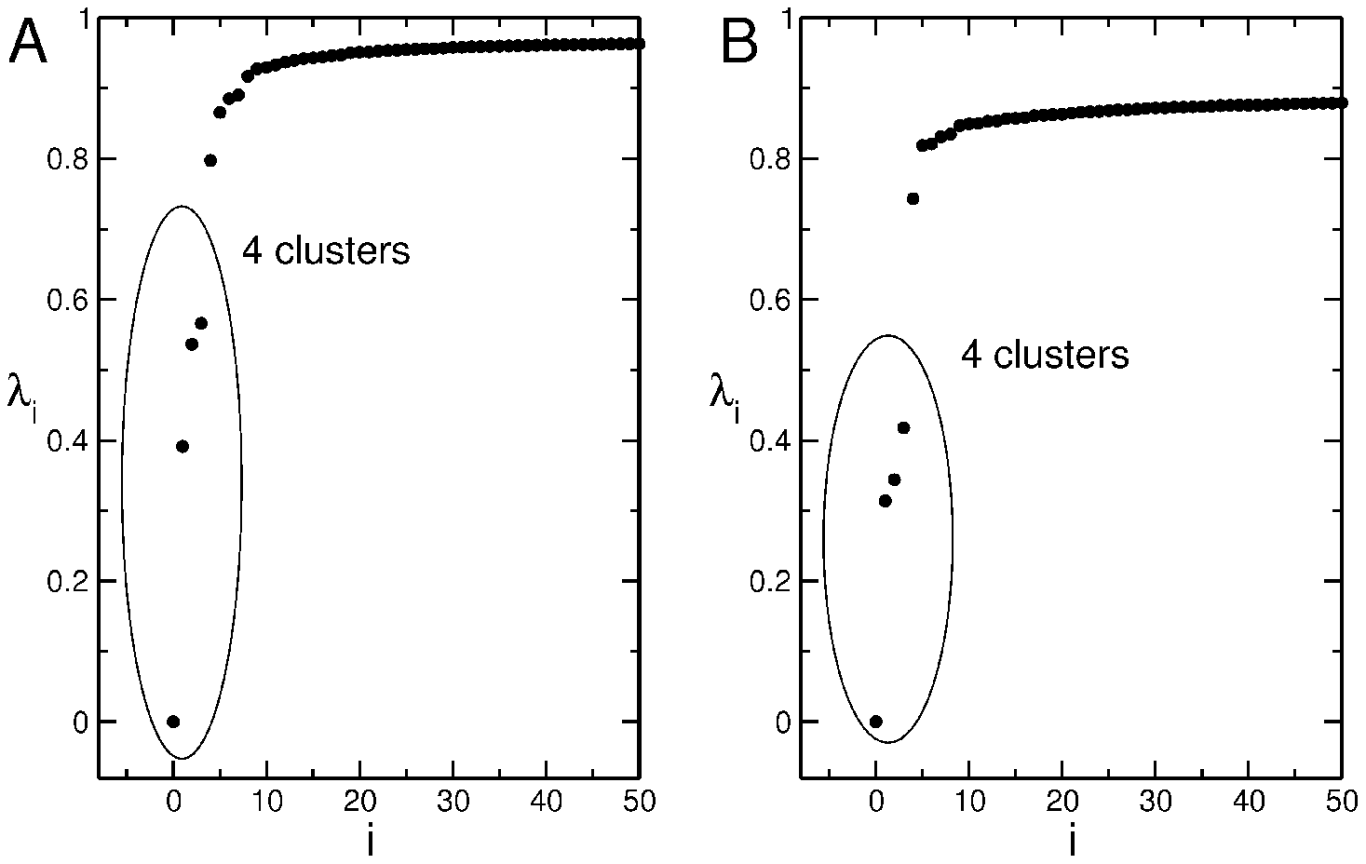
Eigenvalues of the Laplacian matrix. First 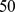 eigenvalues in ascending order of the normalized Laplacian matrix relative to the alignment of 2880 *H. sapiens* promoters. The method used is the Needleman–Wunsch with GAPOPEN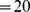 and GAPEXTEND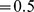 for panel **A**, GAPEXTEND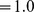 for panel **B**.

Following [73], once an appropriate adjacency matrix is obtained, the first step of the clustering procedure is the determination of the number of clusters. For this purpose, we introduce the normalized Laplacian 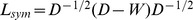 where the degree matrix 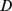 is defined as the diagonal matrix with entries 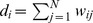. In some particularly successful cases, 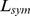 has a block structure, and the multiplicity of its null eigenvalue determines the number of connected components. In real cases, however, data is well mixed, and 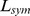 has a unique null eigenvalue corresponding to one connected component, which includes the whole data set. The solution of the problem comes from the matrix perturbation theory [74]. Indeed, given the spectrum 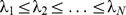 of 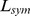, the information about the number of clusters is carried by those eigenvalues wh ich are located close to the null one. The idea is that the actual 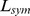 can be read as a perturbation of an *ideal* block matrix, and thus the first 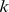 values of the spectrum act as fluctuations of the corresponding null eigenvector of the *ideal* case, with multiplicity 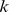. In practice, the more the first 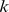 eigenvalues are distant from the others, the more effective will be the separation of data into the 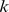 groups. Fig. 7 helps to understand this approach. Both panels show the first part of the spectrum of 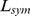 associated to the alignment of 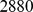 *H. sapiens* promoters with the global algorithm. The first value is zero, and then three consecutive eigenvalues, located far from the others, follow. Accordingly, the resulting number of clusters is 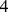. The distance from the fourth eigenvalue to the fifth one is larger in panel B where we used a higher GAPEXTEND value.

#### Clustering algorithm

We are now able to apply the spectral clustering algorithm in order to assign each promoter to one of the clusters. The starting point is the computation of the first 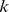 eigenvectors 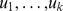 of 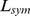, so as to form a new matrix 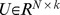 containing the vectors 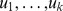 as columns. Let 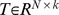 be the matrix obtained from 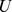 by normalizing the rows to norm 1, namely, 
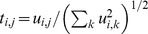
. For 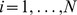 we denote by 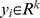 the vector corresponding to the 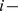
th row of 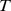. The last point consists in applying the k–means algorithm to the points 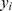 so as to find 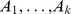 clusters. The iterative procedure of the algorithm works as follows: first, select 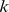 random points as initial centroids. Then, form 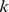 clusters assigning each point 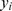 to its closest centroid, according to Euclidean distance. Recompute the centroids as the mean of the points of each cl uster. Repeat until the difference between the centroids coordinates of two consecutive steps reaches a fixed tolerance. For instance, in panel A of Fig. 1 this tolerance was fixed to 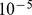.

### Spectral method for identification of regular sequences

Nucleotide sequences in promoters are characterized by the alternation of regular and disordered regions of different length. In particular, the regular ones exhibit various structures, ranging from homogeneous to periodic and palindromic. In this section we describe a method for the identification of all these regular sequences starting from the properties of a mechanical model of the DNA chain. It is worth pointing out that the method is based on a definition of *regularity* of finite–length regions in a promoter, that combines suitable quantitative indicators.

In practice, we adopt the model introduced by Peyrard and Bishop [48]–[50] (see section *Peyrard-Bishop model*). This model simplifies the molecular structure of the DNA by considering only one strand and neglecting the double-helix structure. It takes explicitly into account the nonlinear interactions between the nucleotides and, despite its apparent simplicity, it is quite effective for reproducing the dynamics of DNA at physiological temperatures. For our purposes, it is sufficient to consider the *harmonic* approximation of this model, that is valid in the low–temperature limit. In this sense, what remains of the information contained in the Peyrard-Bishop model are the presence of nearest–neighbor and on–site harmonic interactions and the phenomenological parameters defining their strength (see Eq. (3)). In section *Normal modes* we show that the properties of the chain in the *harmonic* regime are completely determined by the features of the Hessian matrix of the model.

Finally, in section *Determination of regular sequences*, we describe the procedure for the determination of the regular sequences using the eigenvectors of the Hessian matrix.

At variance with the notation adopted for labeling the position of nucleotides in a promoter (namely, 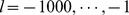), in what follows we adopt the standard numeration for the index 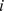 of the sites in an oscillator chain, namely 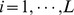 (with 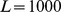 for promoters).

### Peyrard-Bishop model

In the Peyrard-Bishop model each nucleotide 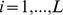 is associated with one degree of freedom 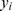, that corresponds to the displacement of the nucleotide from its equilibrium position. This displacement is in the the direction of the hydrogen bonds connecting a nucleotide to its complementary in the opposite strand. The state of the chain is completely determined by the vector 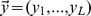. The interaction due to the hydrogen bonds is modeled by a Morse potential. Moreover, the model contains a stacking interaction between nearest neighbor nucleotides: the strength of this interaction decreases when the complementary nucleotides are farther. The total potential energy 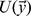 is given by
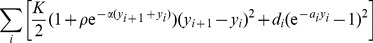
(1)


The parameters 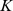, 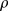 and 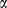 refer to the stacking interactions between two consecutive nucleotides; while the parameters 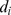 and 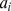 define the depth and the width of the Morse potential, respectively. In order to model heterogeneuos DNA sequences two different values for the couple (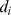, 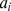) are considered according to the two possible kind of nucleotides, weak (W) and strong (S). The former has two hydrogen bonds, while the latter has three hydrogen bonds. Therefore, the depth for the S Morse potential is chosen 1.5 times the one of the W Morse potential. The model is characterized by a *dichotomic* disorder along the chain: every nucleotide can be associated to the couple of values 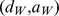 or 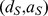. The ground state of the model (i.e., the state of minimal energy) corresponds to a configuration of the chain with 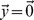. For the promoters analyzed in this paper we have 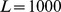, while the parameter set is the one adopted in [75] (in order to avoid convergence problems in the algorithm for the diagonalization of the Hessian matrix of the potential 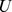 we chose 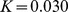 eV/*Å* ^2^ instead of 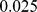 eV/*Å* ^2^).

### Normal modes

The normal modes of the Peyrard–Bishop model of the DNA chain represent small oscillations around the ground state. In order to fully characterize them we need to know the frequencies and the amplitudes of oscillations of every nucleotide (that is equivalent to a harmonic oscillator). A normal mode is in fact a collective motion where every nucleotide vibrates with the same frequency but with a different amplitude. As the chain has L degrees of freedom there are L different ways of oscillation.

#### Approximation of the potential energy

From a mathematical point of view the normal–mode approach corresponds to consider a Taylor series expansion of the potential energy around the minimum 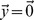. At the second order it reads
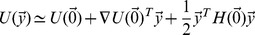
(2)where 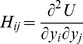 is the symmetric Hessian matrix of the potential energy. Since in the minimum of the potential 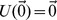 and 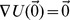, Eq.(2) reduces to
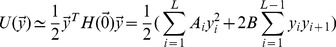
(3)where: 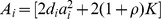 for 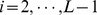, 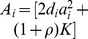 for 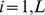 and 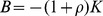. This amounts to the *harmonic* approximation, where the properties of the potential energy are summarized in the Hessian matrix evaluated in the minimum of the potential.

#### Hessian matrix: eigenvalues and eigenvectors

By a suitable change of coordinates 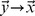, the quadratic form (3) can be rewritten in a diagonal form by a standard procedure (this is done by solving the spectral problem for the Hessian matrix, i.e., 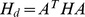 where 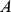 is an orthogonal matrix 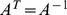, 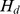 is the Hessian matrix in diagonal form and by setting 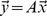). In the new variables, 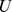 reads as the energy associated to 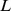 harmonic springs
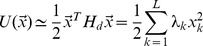
(4)where 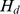 is the diagonal form of the Hessian matrix and 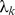 are the eigenvalues.

The eigenvectors 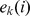 of the Hessian matrix (where 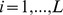 is the nucleotide index relative to the TSS) are the eigenmodes of the DNA chain.

#### Properties of the eigenvectors

Regular sequences in the promoters are recovered by looking at eigenvectors of the Hessian matrix with suitable features of delocalization according to the method described in section *Determination of regular sequences*. In order to apply this procedure, the following indicators have been used to fully characterize the eigenvectors.

1. the *eigenvector center of mass*, 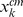, signals the position of the center of the eigenvector along the promoter chain and it is defined by
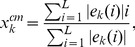
(5)
2. the *eigenvector extension* along the chain is quantified by
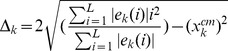
(6)
3. the *eigenvector participation number*, 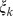, is a measure of the degree of delocalization of the eigenvector and it is defined by
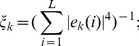
(7)for an eigenvector localized on a single site 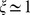, while for a completely delocalized eigenvector 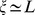 (the eigenvectors are normalized to unity, i.e. 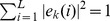).

**Figure 8:**
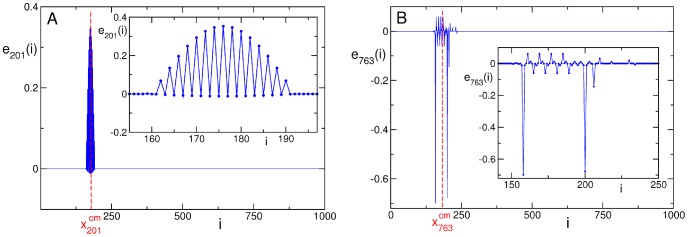
Eigenvectors of the Hessian matrix with different properties of delocalization. The eigenvector 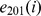, in panel **A**, has comparable values of participation number and extension (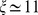 and 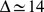), while the eigenvector 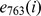, in panel **B**, has a small participation number, 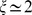, but very large extension (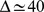). In the insets an enlargement of the region of delocalization is shown. Data refer to the promoter of *H. sapiens* with Entrez GeneID 9542 (the promoter of the neuregulin-2 gene). Entrez Gene is the gene-specific database at the National Center of Biotechnology Information (NCBI) [76].

We want to point out that both the extension and the participation number are necessary to define the properties of the eigenvectors, because the two indicators are not always positively correlated (see Fig. S10). In fact, for some eigenvectors the degree of delocalization essentially coincides with the extension of the eigenvector (see panel A of Fig. 8). On the other hand, there are eigenvectors having very small participation number despite the very large extension, and this is typically due to the presence of very large components on a few sites and much smaller components on many sites in between (see panel B of Fig. 8).

### Determination of regular sequences

By regular sequence we mean a region of a promoter that exhibits any spatial regularity in the *weak-strong* binary code. Eigenvectors with large enough degree of delocalization, determined by the participation number 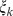, generally extend over regular regions. Accordingly, the method for the identification of the regular sequences, that we are going to describe in detail, needs from the very beginning a conventional definition of *delocalized* eigenvectors and of their *effective* extension along the sequence (criteria I and II).

**Figure 9:**
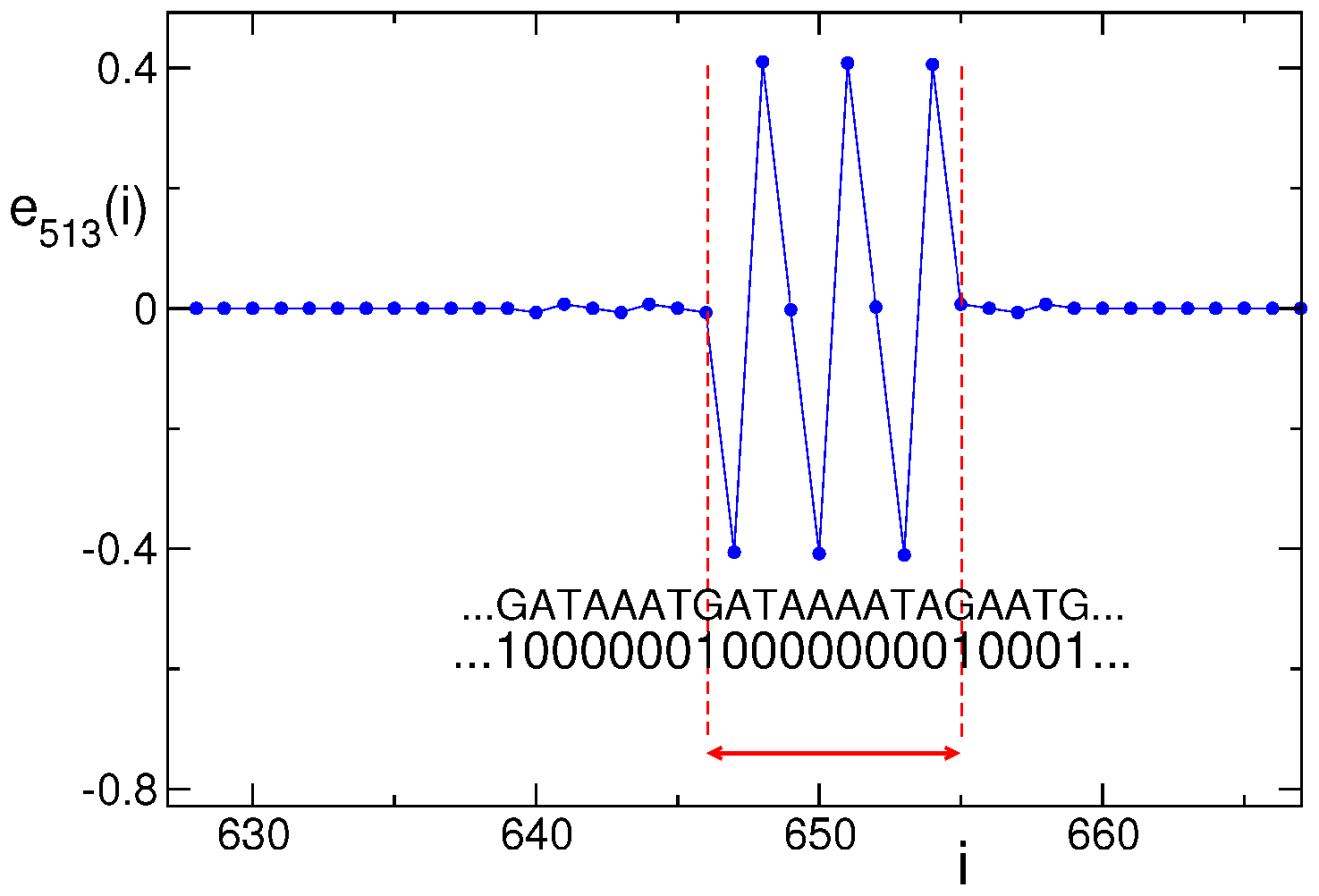
Start site and end site of an eigenvector. Determination of the effective extension (region in between the dashed lines) of a *delocalized* eigenvector overlying regular sequences. Notice the very small components of the eigenvectors aside the regular region. A portion of the sequence is reported both in quaternary and in binary code. Data refer to the promoter of *H. sapiens* with Entrez GeneID 54808 (the promoter of the dymeclin gene).

- We consider *delocalized* those eigenvectors with participation number exceeding a fixed threshold value, i.e. 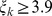, that typically correspond to a region of at least 7 nucleotides. This *heuristic* choice is justified by the fact that many regular motifs of biological interest correspond to such a size (e.g, the TATA–box, that contains 8 nucleotides).
- The start-site, 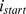, and end-site, 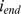, of a delocalized eigenvector are identified according to the following conditions,
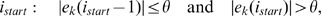


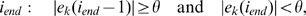
with 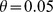. The heuristic choice of the value of the threshold 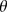 allows to remove the ambiguity that can be introduced by very small components of the eigenvectors (see Fig. 9).

Moreover, we use the property that the eigenvectors of an isolated regular region overlap with the eigenvectors of the whole promoter in that region.

A regular region of the promoter composed of 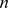 nucleotides has exactly 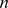 eigenvectors and if we could ideally neglect border effects also the whole promoter would have 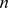 eigenvectors extending over the regular region. Actually, in practical cases this condition on the number of the eigenvectors of the whole promoter can be only approximatively satisfied. This technical point is discussed in file Text S1 (see also Fig. S9).

Therefore, the procedure for the determination of regular sequences is summarized in the following steps:

1. identification of the start-site and of the end-site for all the *delocalized* eigenvectors (see criteria I and II);
2. determination of the number of eigenvectors between the start-site and the end-site and comparison with the number of nucleotides contained in the same region: these quantities are assumed to be equivalent within a 30% tolerance.

**Figure 10:**
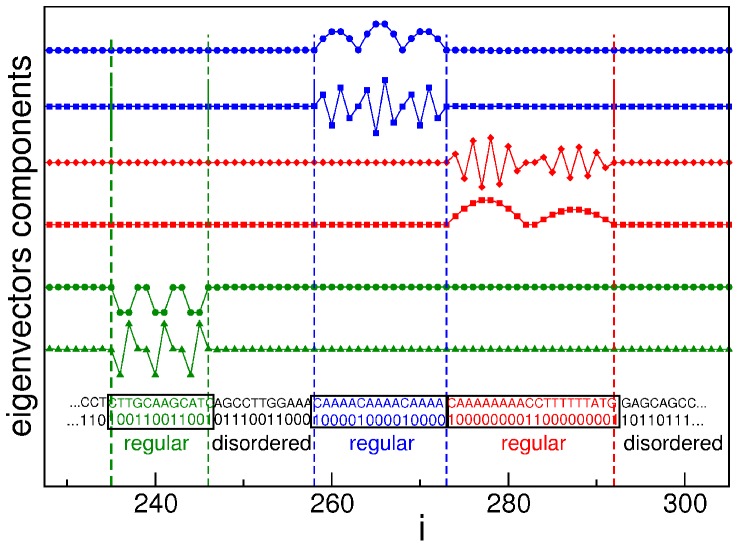
Regular and disordered sequences of a promoter. The regular sequences (highlighted in the black frames) are determined by the *delocalized* eigenvectors of the Hessian matrix. For the sake of clarity, for each of the three examples shown here we report just two of the eigenvectors, whose total number is 10 (green case), 16 (blue case) and 16 (red case). The sequence of the promoter is reported both in quaternary and in binary code. The curves refer to eigenvectors n. 988, 577, 567, 998, 946, 627 (resp. from the top to the bottom) of promoter with Entrez GeneID 9542 of *H. sapiens*.

In Fig. 10 we show some examples of regular sequences determined by delocalized eigenvectors. Following this procedure we were able to rule out false identifications.

### Repeat masker

Trasposons were identified by RepeatMasker [67], version 3.3.0, a program that screens DNA sequences for interspersed repeats. The output of the program is a detailed annotation of the repeats that are present in the query sequence. The options were chosen as follows:

Search engine: abblast

Speed/sensitivity: Default

DNA source: Human for *H. sapiens*, Mammal for *P. troglodytes*, Mouse for *M. musculus*, Danio for *D. rerio*, Arabidopsis thaliana for *A. thaliana*.

Comparison species: none

Alignment options: no alignments returned

Masking options: Repetitive sequences in lower case

Contamination check: No contamination check

Repeat options: Don't mask simple repeats or low complexity DNA

Artifact check: Report E. coli IS artifacts

Matrix: RepeatMasker choice

Divergence cutoff: none

## Supporting Information

Figure S1
**BCA of each of the clusters obtained with the clustering algorithm for *P. troglodytes* (panel A) and *M. musculus* (panel B).** We report the frequency 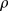 of each of the four nucleotides A (black), T (blue), C (red) and G (green) as a function of the position 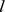 along the promoter (0 corresponds to the TSS).(TIFF)

Figure S2
**BCA of each of the clusters obtained with the clustering algorithm for *D. rerio* (panel A) and *A. thaliana* (panel B).** We report the frequency 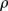 of each of the four nucleotides A (black), T (blue), C (red) and G (green) as a function of the position 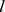 along the promoter (0 corresponds to the TSS). Note that alignment and clustering are performed taking into account only 100 nucleotides before the TSS.(TIFF)

Figure S3 **The most frequent regular sequences found in the clusters of *P. troglodytes* (panel A) and *M. musculus* (panel B).** We report the percentage of promoters of the cluster in which the sequence appears at least once (left column), and the percentage of times the sequence is found inside a transposon (right column): it is calculated dividing the number of times it appears in a transposon by the total number of times it appears in the cluster.
(TIFF)

Figure S4 **The most frequent regular sequences found in the entire sample of 2880 promoters of *D. rerio* (panel A) and *A. thaliana* (panel B).** We report the percentage of promoters in which the sequence appears at least once (left column), and the percentage of times the sequence is found inside a transposon (right column): it is calculated dividing the number of times it appears in a transposon by the total number of times it appears in the cluster.
(TIFF)

Figure S5 **Distribution of the different families of transposons in the four clusters of** ***P. troglodytes*** **.** We report the total percentage of nucleotides in the cluster covered by transposons (pie chart) and the percentage of nucleotides covered by each family of transposons (histogram). Note the different scales in the histograms.
(TIFF)

Figure S6 **Distribution of the different families of transposons in the four clusters of** ***M. musculus*** **.** It is shown the total percentage of nucleotides in the cluster covered by transposons (pie chart) and the percentage of nucleotides covered by each family of transposons (histogram). Note the different scales in the histograms.
(TIFF)

Figure S7 **Distribution of the different families of transposons in the four clusters of** ***D. rerio*** **.** It is shown the total percentage of nucleotides in the cluster covered by transposons (pie chart) and the percentage of nucleotides covered by each family of transposons (histogram).
(TIFF)

Figure S8 **Distribution of the different families of transposons in the two clusters of** ***A. thaliana*** **.** It is shown the total percentage of nucleotides in the cluster covered by transposons (pie chart) and the percentage of nucleotides covered by each family of transposons (histogram).
(TIFF)

Figure S9
**Participation number vs. center of mass.** Participation number, 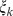, as a function of the eigenvector center of mass, 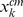, for the whole promoter, (red) squares, and for an isolated region of the promoter composed of the first 200 nucleotides, (green) triangles. Data refer to the promoter of *H. sapiens* with Entrez GeneID 9542.(TIFF)

Figure S10
**Eigenvector extension,** 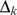 **, as a function of the participation number,** 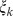 **.** The (red) dashed circles refer to eigenvectors with different properties of localization. The eigenvector 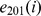 (see Fig. 8 in Methods) has comparable values of 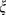 and 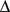. While 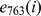 (see Fig. 8 in Methods) has a small participation number, 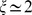, but large extension (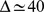). Data refer to the promoter of *H. sapiens* with Entrez GeneID 9542.(TIFF)

Figure S11
**CG content and CpG islands.** We report dinucleotide density 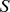 as a function of the position along the promoter (0 corresponds to the TSS). Data are obtained analysing the promoters of C1.(TIFF)

Figure S12
**Histogram of the length distribution of the regular sequences in the clusters of** ***H. sapiens*** **.** We report the frequency of each length 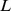 as a function of 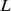.(TIFF)

Text S1 **Additional remarks on Supporting Information.**
(PDF)

Table S1 **Number of regular sequences.** We report the total number of regular sequences found in the clusters of *H. sapiens* (first row) and the number of the corresponding distinct sequences (second row).
(JPG)
