# Supplementary figures and images for: Genome-Wide Analysis of Promoters: Clustering by Alignment and Analysis of Regular Patterns
Source: PLoS One. 2014 Jan 22;9(1):e85260. doi: 10.1371/journal.pone.0085260 (PMC3898993; doi:10.1371/journal.pone.0085260)

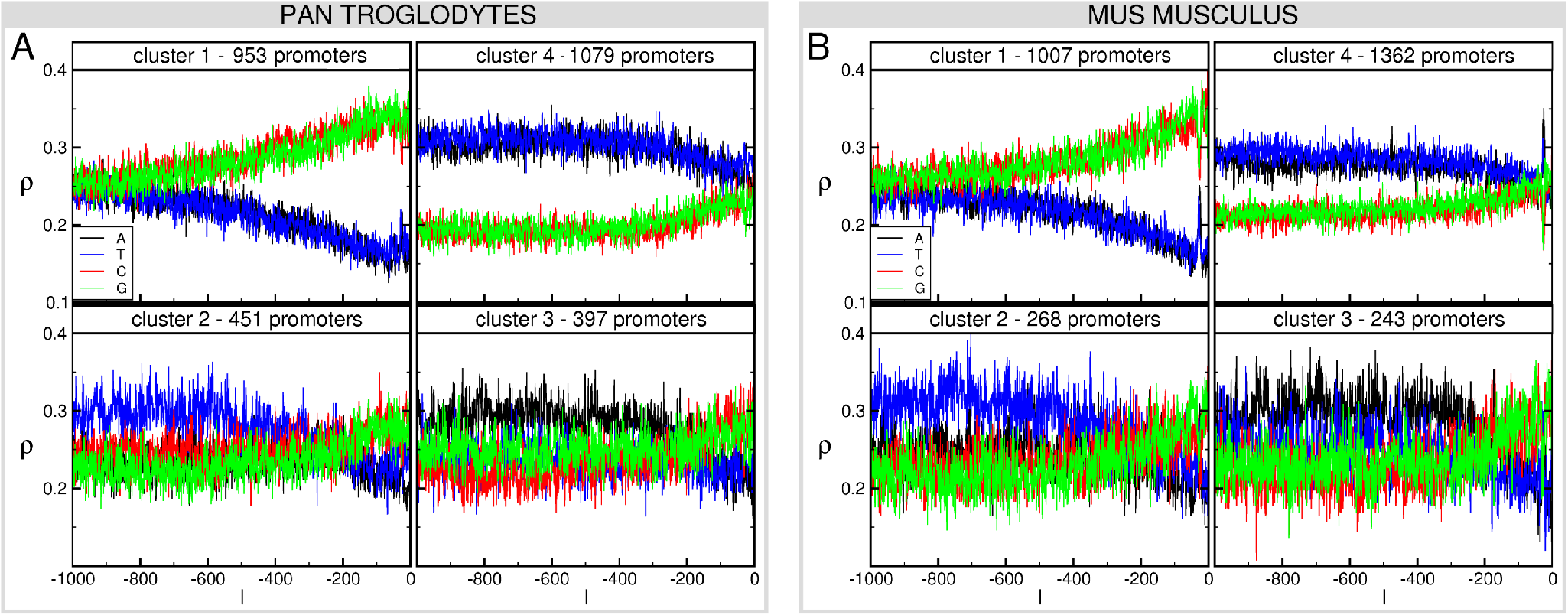

Supplement: Figure S1 — BCA of each of the clusters obtained with the clustering algorithm for P. troglodytes (panel A) and M. musculus (panel B). We report the frequency of each of the four nucleotides A (black), T (blue), C (red) and G (green) as a function of the position along the promoter (0 corresponds to the TSS). (TIFF) [file pone.0085260.s001.tiff]

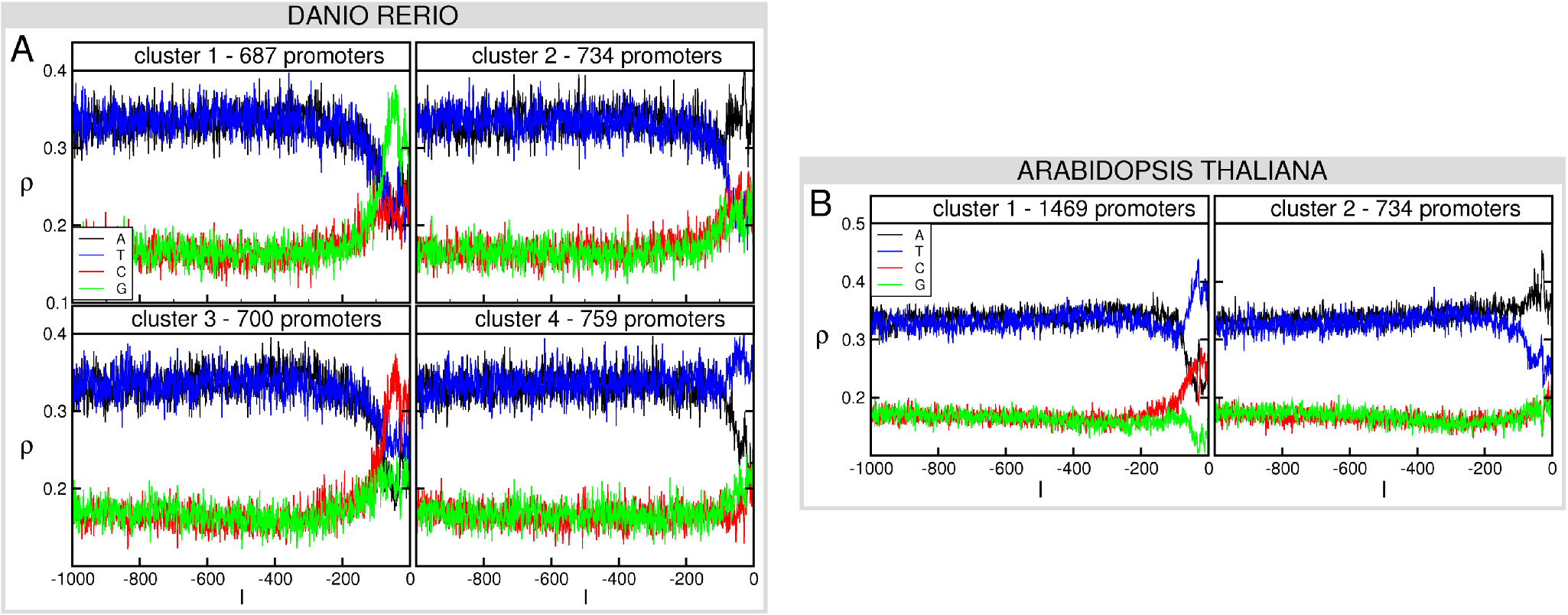

Supplement: Figure S2 — BCA of each of the clusters obtained with the clustering algorithm for D. rerio (panel A) and A. thaliana (panel B). We report the frequency of each of the four nucleotides A (black), T (blue), C (red) and G (green) as a function of the position along the promoter (0 corresponds to the TSS). Note that alignment and clustering are performed taking into account only 100 nucleotides before the TSS. (TIFF) [file pone.0085260.s002.tiff]

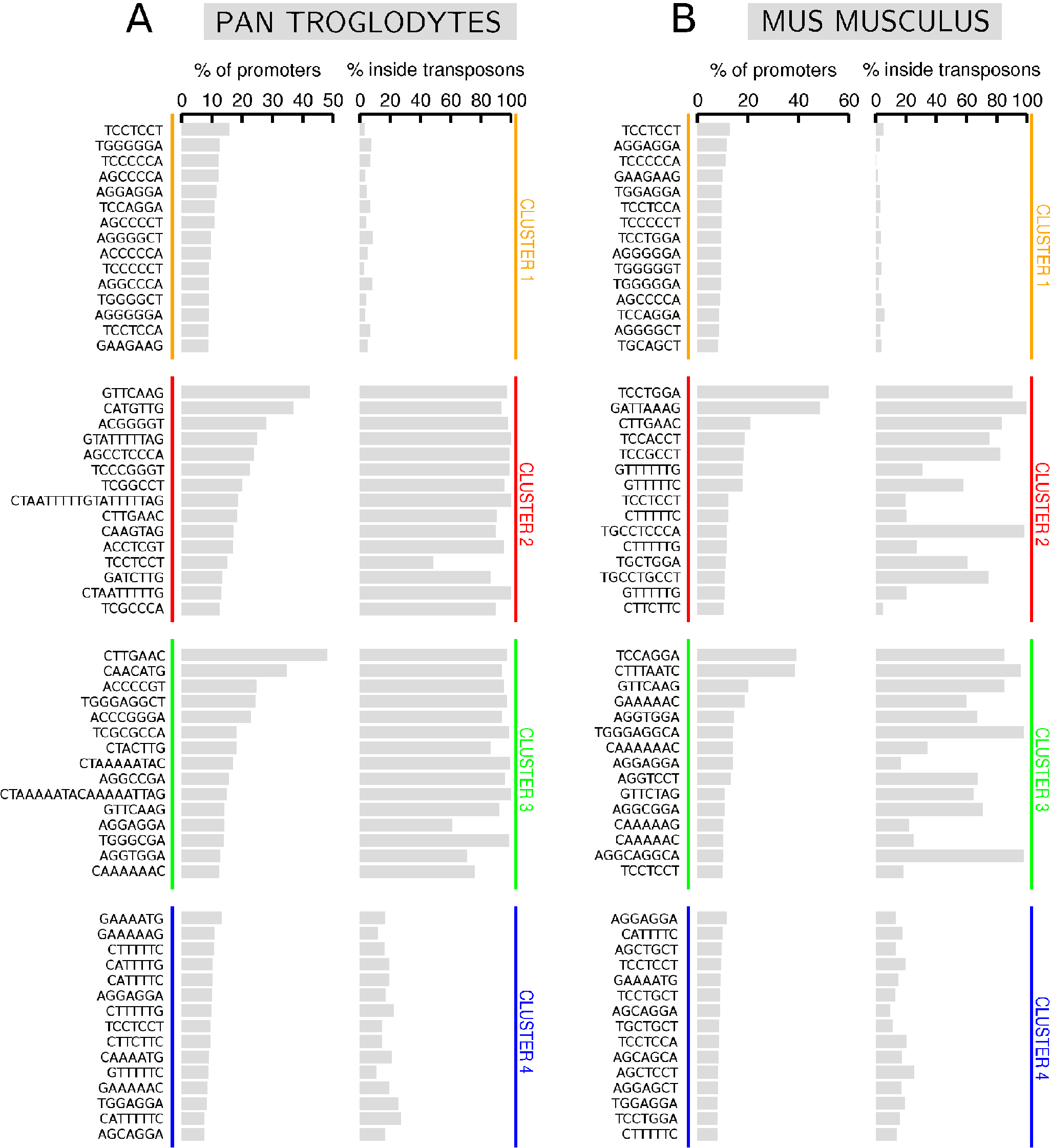

Supplement: Figure S3 — The most frequent regular sequences found in the clusters of P. troglodytes (panel A) and M. musculus (panel B). We report the percentage of promoters of the cluster in which the sequence appears at least once (left column), and the percentage of times the sequence is found inside a transposon (right column): it is calculated dividing the number of times it appears in a transposon by the total number of times it appears in the cluster. (TIFF) [file pone.0085260.s003.tiff]

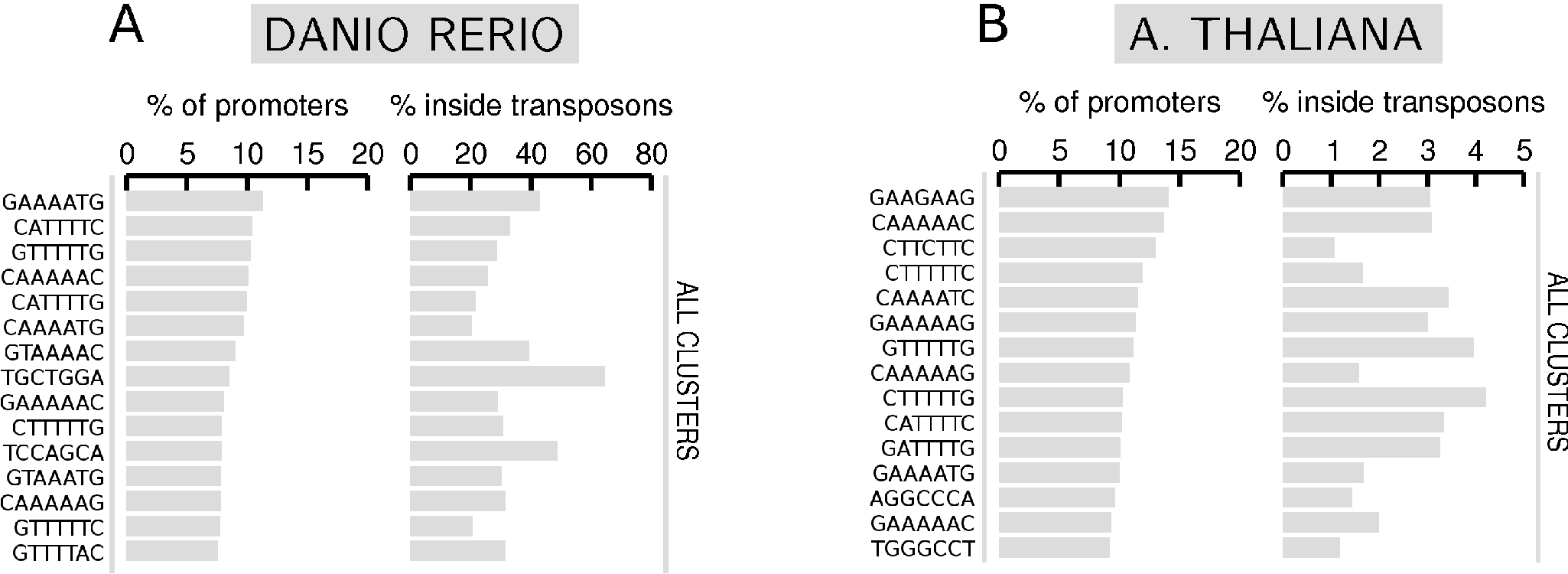

Supplement: Figure S4 — The most frequent regular sequences found in the entire sample of 2880 promoters of D. rerio (panel A) and A. thaliana (panel B). We report the percentage of promoters in which the sequence appears at least once (left column), and the percentage of times the sequence is found inside a transposon (right column): it is calculated dividing the number of times it appears in a transposon by the total number of times it appears in the cluster. (TIFF) [file pone.0085260.s004.tiff]

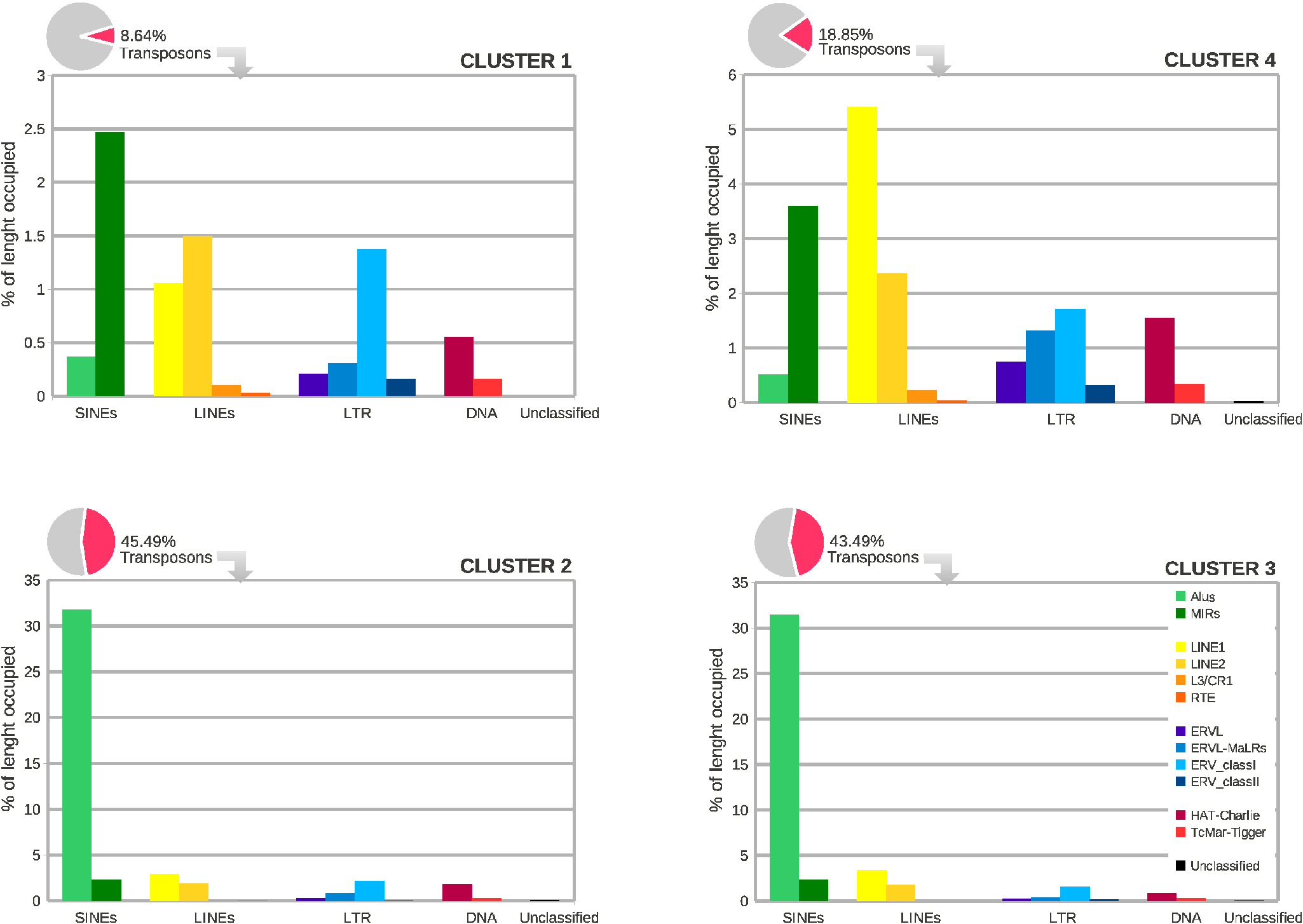

Supplement: Figure S5 — Distribution of the different families of transposons in the four clusters of P. troglodytes . We report the total percentage of nucleotides in the cluster covered by transposons (pie chart) and the percentage of nucleotides covered by each family of transposons (histogram). Note the different scales in the histograms. (TIFF) [file pone.0085260.s005.tiff]

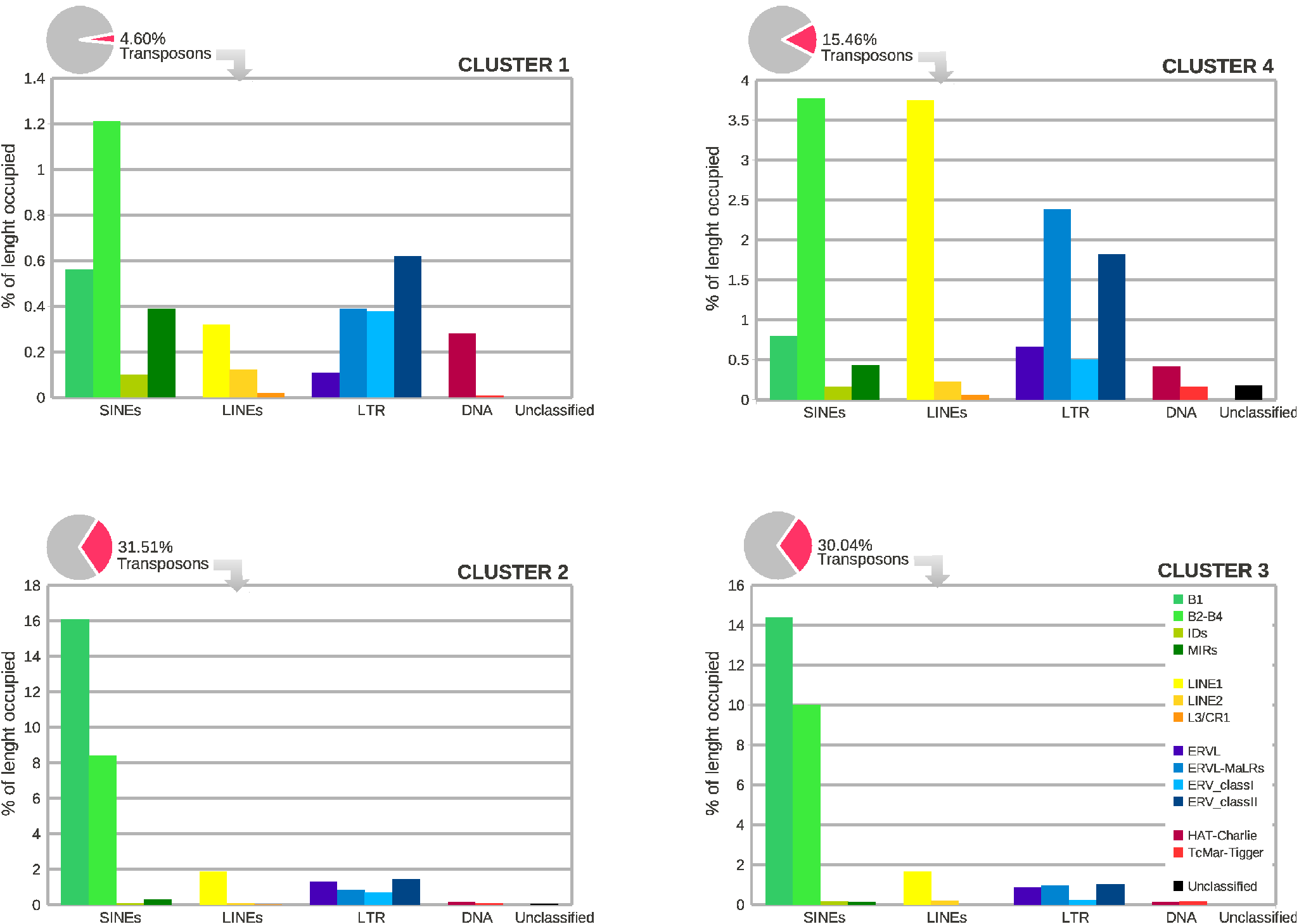

Supplement: Figure S6 — Distribution of the different families of transposons in the four clusters of M. musculus . It is shown the total percentage of nucleotides in the cluster covered by transposons (pie chart) and the percentage of nucleotides covered by each family of transposons (histogram). Note the different scales in the histograms. (TIFF) [file pone.0085260.s006.tiff]

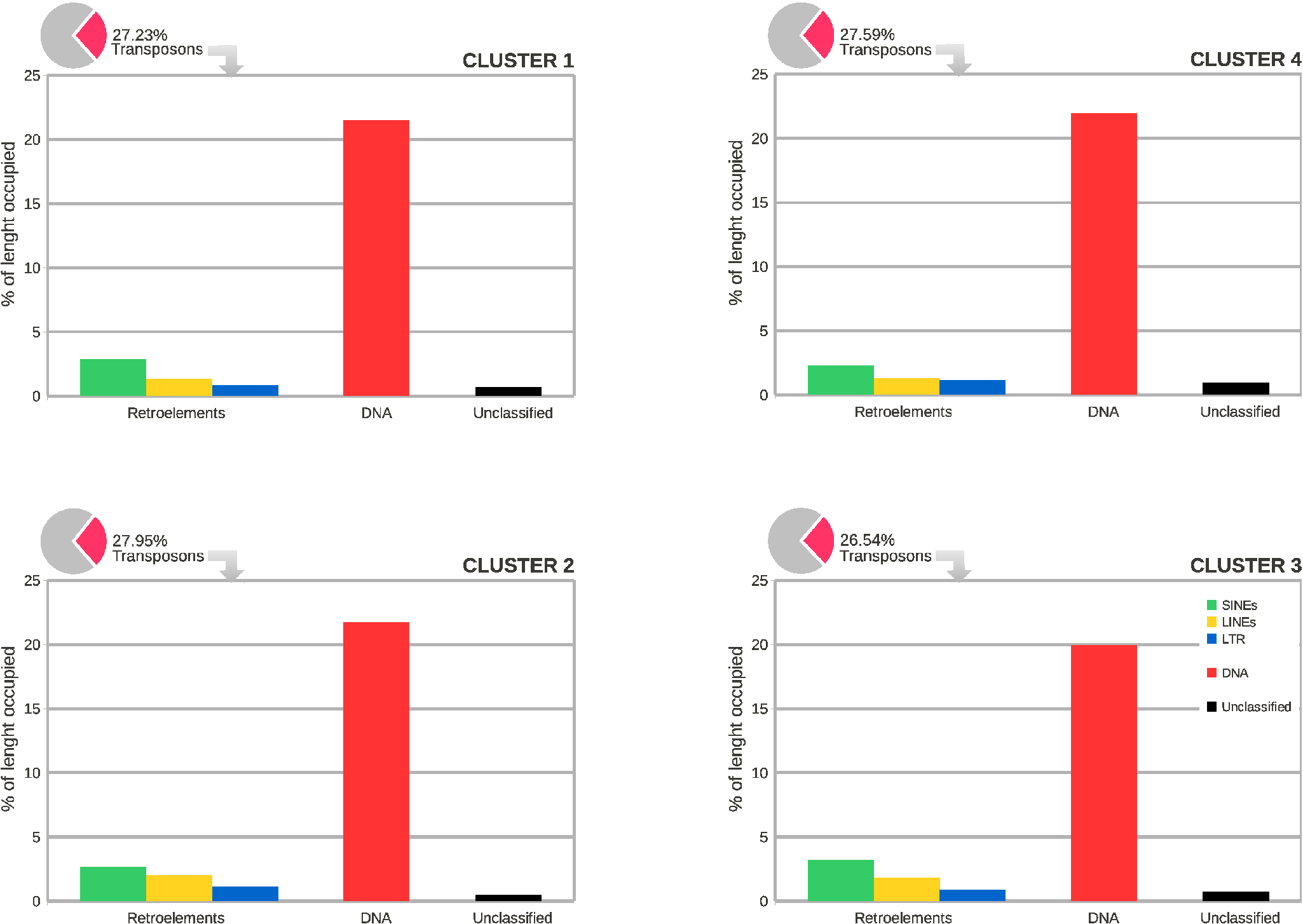

Supplement: Figure S7 — Distribution of the different families of transposons in the four clusters of D. rerio . It is shown the total percentage of nucleotides in the cluster covered by transposons (pie chart) and the percentage of nucleotides covered by each family of transposons (histogram). (TIFF) [file pone.0085260.s007.tiff]

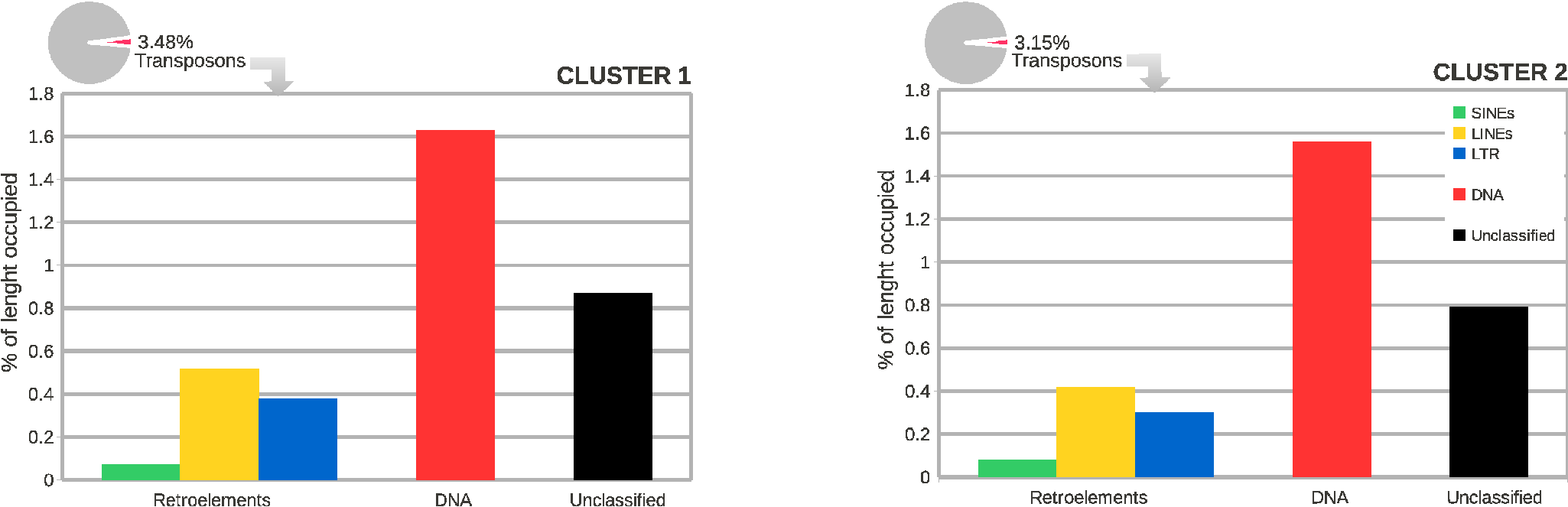

Supplement: Figure S8 — Distribution of the different families of transposons in the two clusters of A. thaliana . It is shown the total percentage of nucleotides in the cluster covered by transposons (pie chart) and the percentage of nucleotides covered by each family of transposons (histogram). (TIFF) [file pone.0085260.s008.tiff]

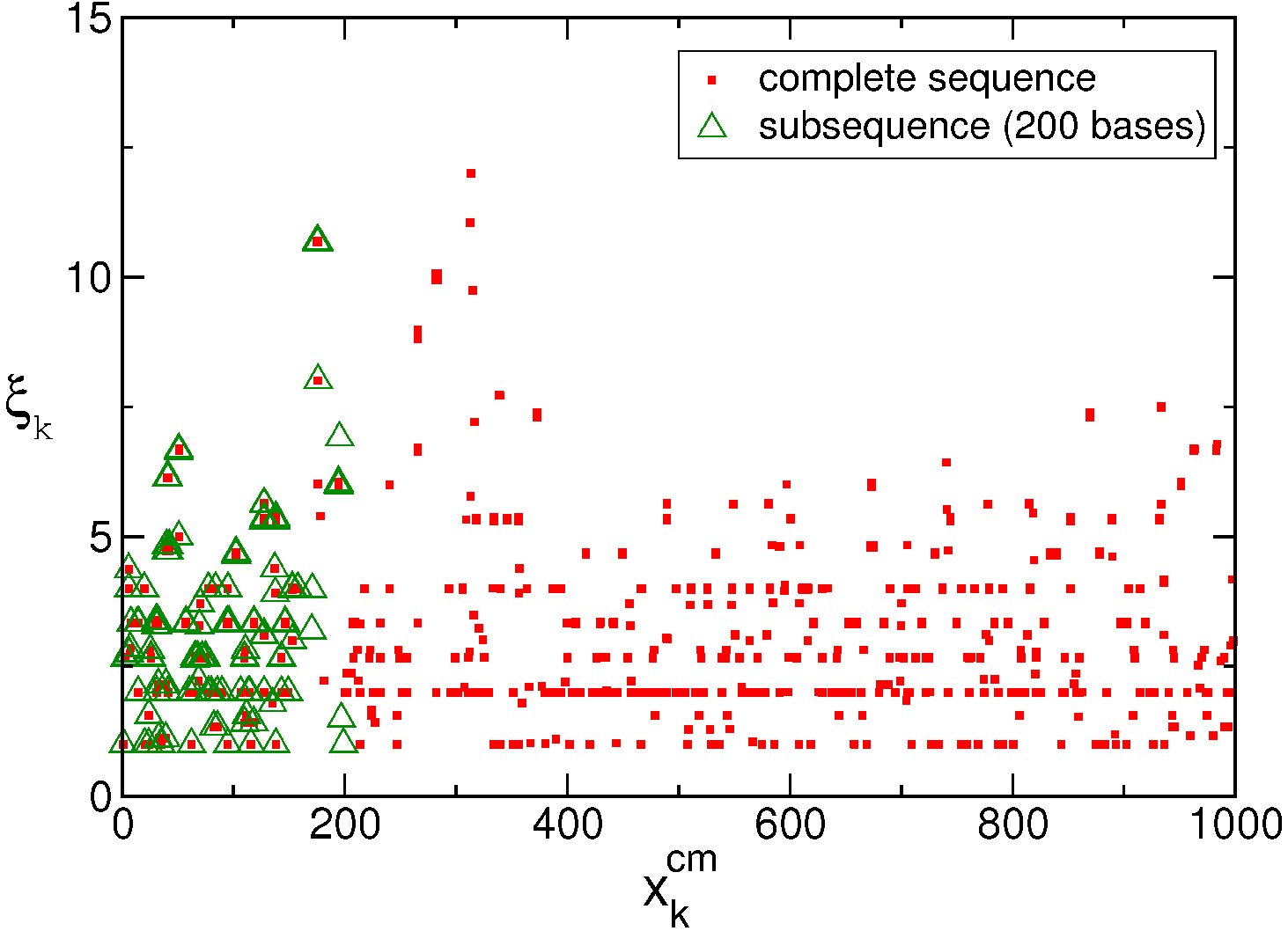

Supplement: Figure S9 — Participation number vs. center of mass. Participation number, , as a function of the eigenvector center of mass, , for the whole promoter, (red) squares, and for an isolated region of the promoter composed of the first 200 nucleotides, (green) triangles. Data refer to the promoter of H. sapiens with Entrez GeneID 9542. (TIFF) [file pone.0085260.s009.tiff]

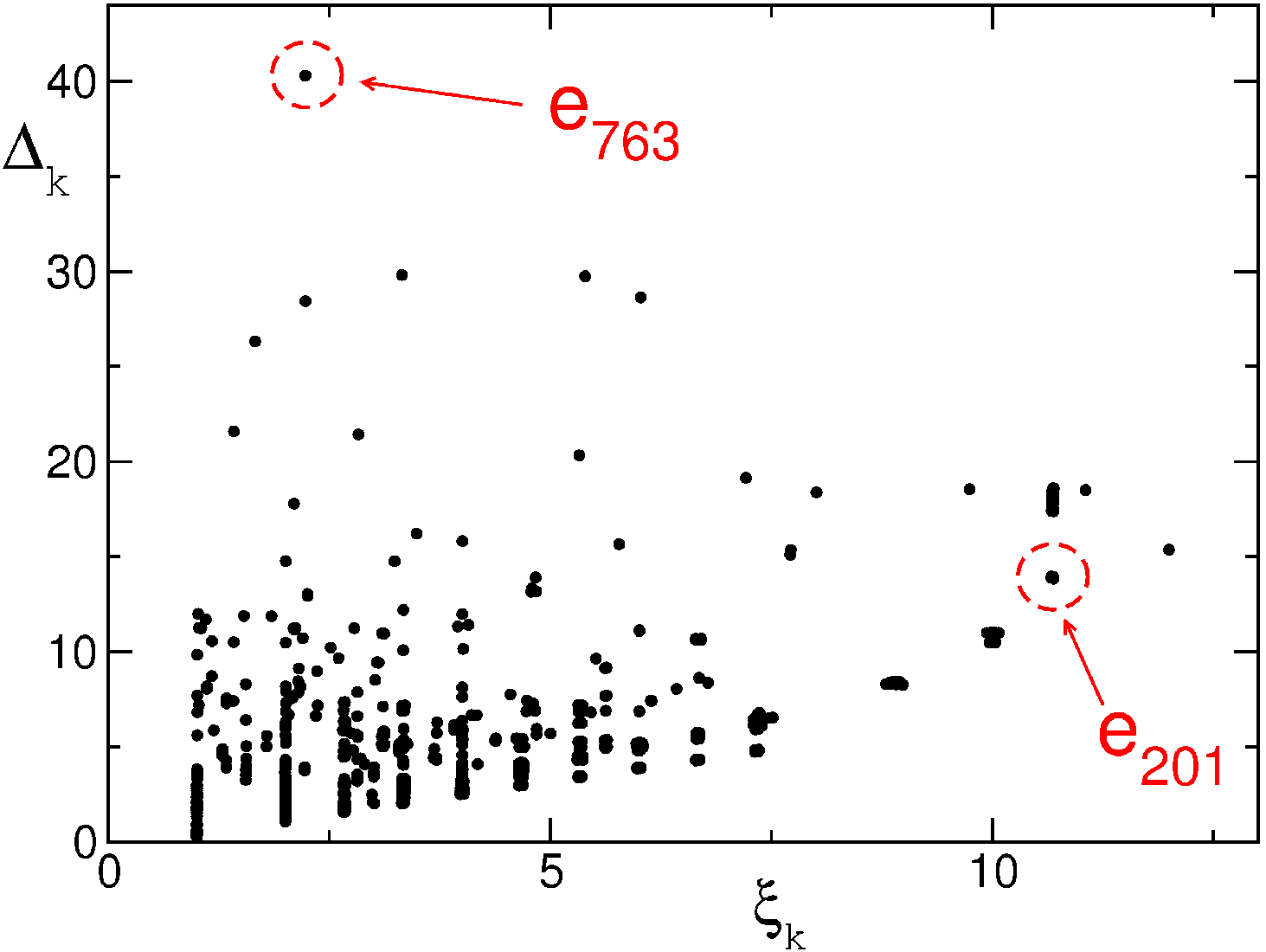

Supplement: Figure S10 — Eigenvector extension, , as a function of the participation number, . The (red) dashed circles refer to eigenvectors with different properties of localization. The eigenvector (see Fig. 8 in Methods) has comparable values of and . While (see Fig. 8 in Methods) has a small participation number, , but large extension (). Data refer to the promoter of H. sapiens with Entrez GeneID 9542. (TIFF) [file pone.0085260.s010.tiff]

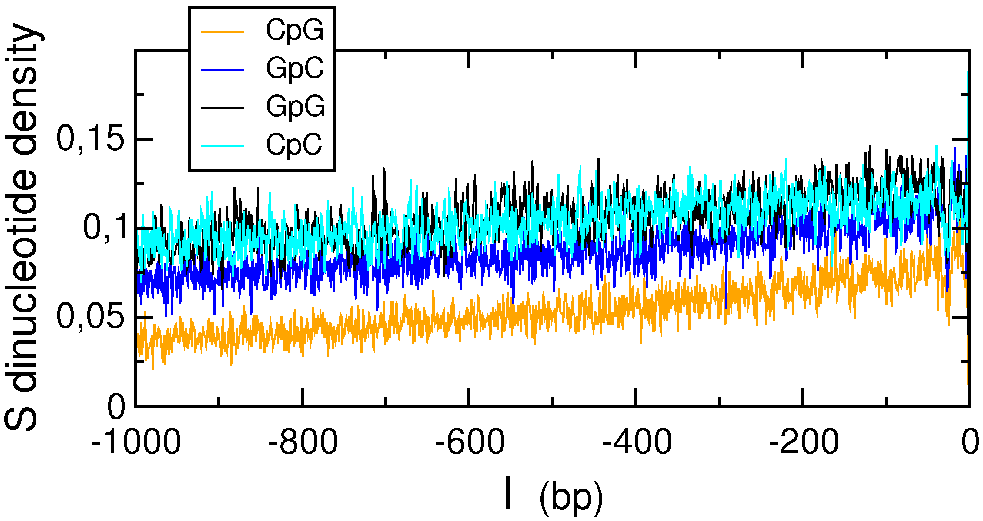

Supplement: Figure S11 — CG content and CpG islands. We report dinucleotide density as a function of the position along the promoter (0 corresponds to the TSS). Data are obtained analysing the promoters of C1. (TIFF) [file pone.0085260.s011.tiff]

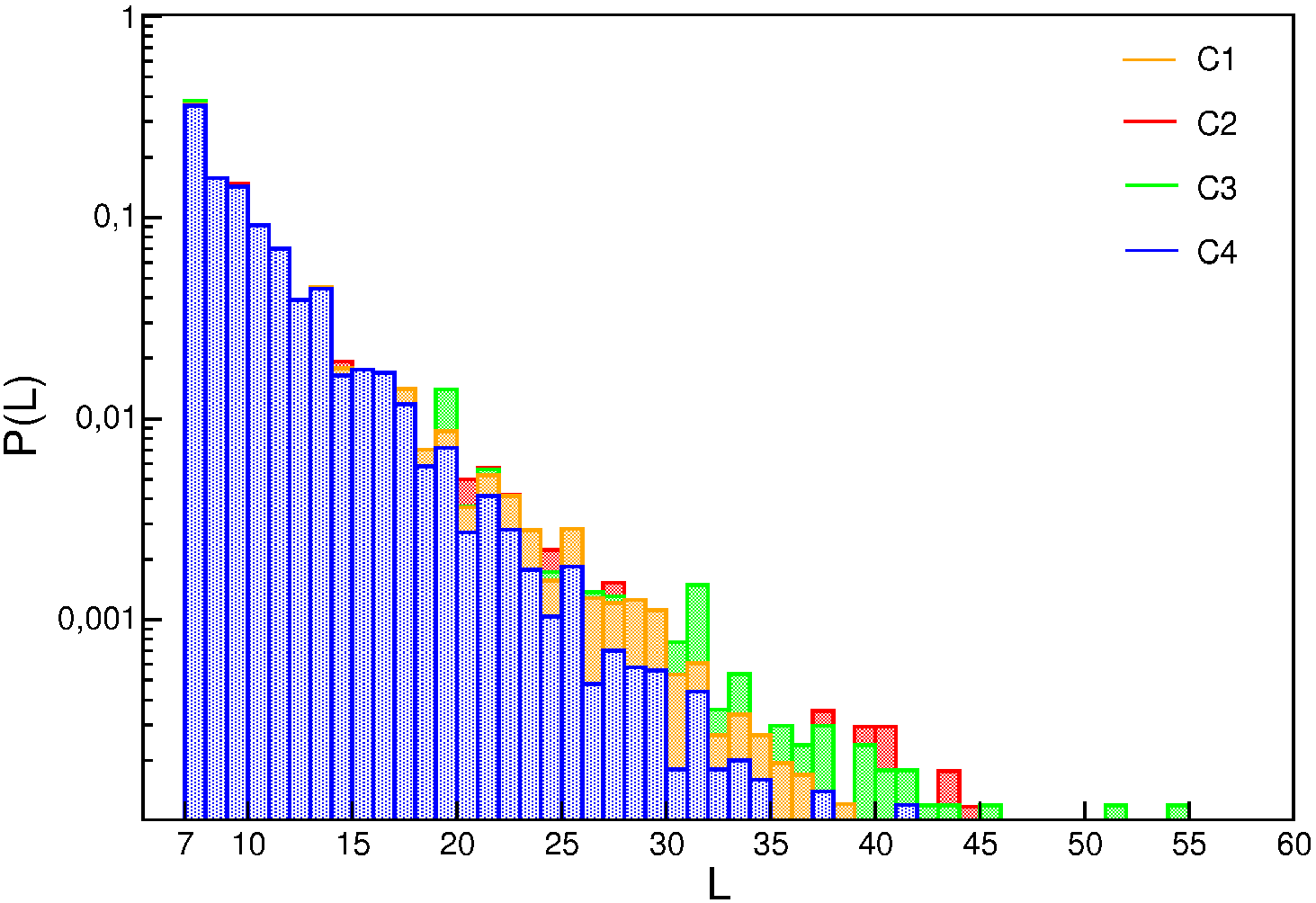

Supplement: Figure S12 — Histogram of the length distribution of the regular sequences in the clusters of H. sapiens . We report the frequency of each length as a function of . (TIFF) [file pone.0085260.s012.tiff]

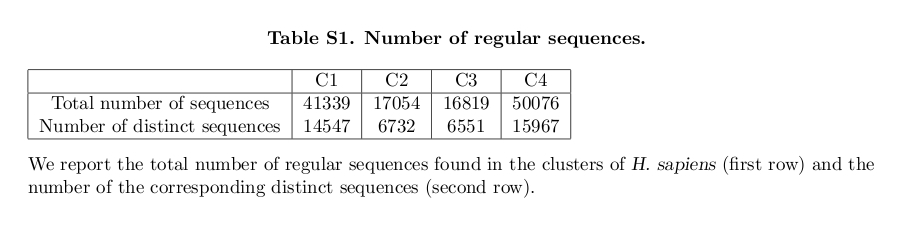

Supplement: Table S1 — Number of regular sequences. We report the total number of regular sequences found in the clusters of H. sapiens (first row) and the number of the corresponding distinct sequences (second row). (JPG) [file pone.0085260.s014.jpg]
