# Supplementary material for: Genome-Wide Analysis of Promoters: Clustering by Alignment and Analysis of Regular Patterns
Source: PLoS One. 2014 Jan 22;9(1):e85260. doi: 10.1371/journal.pone.0085260 (PMC3898993; doi:10.1371/journal.pone.0085260)
Supplement: Text S1 — Additional remarks on Supporting Information. (PDF) [file pone.0085260.s013.pdf]

Supplementary Materials

Clustering and BCA of other species

We report here the results obtained for *P. troglodytes*, *M. musculus*, *D. rerio* and *A. thaliana*, making use of the same clustering procedure adopted for *H. sapiens*.

The first two mammalian species exhibit four clusters as found for *H. sapiens*. The BCA of samples made by 2880 promoters for both species are reported in Fig. S1. The similarity with *H. sapiens* is evident, thus suggesting that the clustering procedure singles out quite universal global properties of mammalian promoters.

On the other hand, this clustering procedure does not work for *D. rerio* and *A. thaliana*: the alignment algorithm applied to the entire promoter extension does not allow to identify different clusters. In fact, according to the results reported in [15, 34], this is not an unexpected result. BCA as well as entropic indicators show that in these species the region affected by some functional constraints of promoters reduces to a portion of the whole sequence close to the TSS, typically extending over 100 nucleotides. Accordingly, for *D. rerio* and *A. thaliana* we have applied the alignment algorithm to this shorter and certainly more specialized region of the promoter. With such a recipe we have obtained again clear signatures of different promoter clusters. For instance, in Fig. S2 we show that a sample of 2880 promoters of *D. rerio* yields four clusters of almost equal size, dominated in the last 100 nucleotides by A, T, C and G nucleotides, respectively. For what concerns *A. thaliana*, we have obtained just two clusters (see Fig. S2) whose BCA exhibits significantly different features only in the region close to the TSS, where either A or T nucleotides dominate.

The most frequent regular sequences in *P. troglodytes*, *M. musculus*, *D. rerio* and *A. thaliana*

Fig. S3 contains the list of the 15 most frequent regular sequences found in each cluster of *P. troglodytes* and *M. musculus*. The left column shows the percentage of promoters in each cluster that contain the sequence at least once. We have observed that, in most cases, any sequence is found inside a promoter only once. Accordingly, the sequences contained in a large percentage of promoters are also the most frequent ones. The large majority of the regular sequences in *P. troglodytes* coincide with those of *H. sapiens*, while the most frequent sequences of *M. musculus* are quite different from those of these two mammalian species.

After having computed how many times each regular sequence appears in all the promoters of each cluster, in the right column of Fig. S3 we report the fraction of times it is contained inside a transposon. We find evidence of a strong correlation between the most frequent regular sequences of C2 and C3 and transposons.

In *D. rerio* and *A. thaliana*, the search for regular sequences has been performed in all of the 1000 nucleotides of each promoter, even if the clusters differentiate only in the 100 nucleotides upstream the TSS. We have found that, at variance with mammals, the most common regular sequences are typically the same in all the clusters. Accordingly, in Fig. S4 we report the data of the 15 most common regular sequences found in whole sample of 2880 promoters.

Transposons

In this section we report the results obtained with the Repeat Masker software [67] (see Methods), that screens DNA sequences for transposons. We have identified transposons in the clusters of *P. troglodytes*, *M. musculus*, *D. rerio* and *A. thaliana*.

The transposon content of each cluster and the percentage of the most frequent family of transposons for *P. troglodytes* are very close to those obtained for *H. sapiens* (Fig. S5). Some similarities with

H. sapiens and *P. troglodytes* still emerge in *M. musculus* (Fig. S6). As in *H. sapiens*, we have observed that the regular sequences in C2 and C3 of *P. troglodytes* (*M. musculus*) are mostly related with Alu elements (B1 elements). This aspect reflects both the conserved features of the old Alu families which spread among the mammalian genome before the primate-rodent split about 80 million year ago and the more recent primate-specific and murine-specific features acquired after their divergence [76].

On the other hand, in *D. rerio* and *A. thaliana* we do not observe differences in transposon content among the clusters (Fig. S7 and Fig. S8). Nonetheless, we find that regular sequences and transposons are definitely less correlated in *D. rerio* than in mammalian species. As far as *A. thaliana* is concerned such a correlation is even weaker (see Fig. S4).

Properties of the eigenvectors of the Hessian matrix

In section *Spectral method for identification of regular sequences* of Methods we showed how to recover all the regular sequences in the promoters by looking at delocalized eigenvectors of the Hessian matrix of a mechanical model of the DNA chain. In this section we report additional details concerning the characterization of the properties of the eigenvectors and of the regular sequences.

Fig. S9 shows the participation number ξ_k, as a function of the center of mass, x_k^{cm}, for all the eigenvectors ($k = 1, \cdots, L$). One can observe that all eigenvectors have a limited degree of delocalization, ranging from a few sites to tens of sites for the most extended eigenvectors (i.e. $\xi_k < L$). The participation number ξ_k (i.e., the degree of delocalization of the eigenvectors) is essentially uncorrelated with the position along the promoter. Similarly, the center of mass of the eigenvectors, x_k^{cm}, is uniformly distributed over the entire promoter extension.

It is worth outlining also the different properties of delocalization of the eigenvectors. Fig. S10 shows the typical relation between the extension, Δ_k, and the participation number, ξ_k, for all the eigenvectors. From the figure it appears that a large extension is not always correlated with a large delocalization: there are eigenvectors having $\xi \simeq \Delta$ but also eigenvectors with considerably different values of the two indicators. Two typical instances of this different behavior are signaled in the figure by a dashed circle and correspond to the eigenvectors $e_{201}(i)$ and $e_{763}(i)$ reported in Fig. 8 of Methods.

A remarkable property used in the determination of regular sequences is that the eigenvectors corresponding to any isolated region of the promoter overlap to a large extent the eigenvectors corresponding to the entire promoter in that region. This is summarized in a compact form in Fig. S9, where a comparison between the features of the eigenvectors of the whole sequence and of a region composed of the first 200 nucleotides is shown: the two patterns completely overlap, apart a few unavoidable mismatches due to border effects in the region around the end of the sequence.

CpG dinucleotide analysis

A common explanation of the GC rise in mammalian promoters is the presence of the so-called CpG islands, i.e. GC-rich regions of DNA (typically 0.5-2 kb in length) that are relatively enriched in CpG dinucleotides with respect to the rest of the genome [77,78]. In a previous work [15] it was addressed the question if the patterns observed in TATA-less sequences (those corresponding to C1 in this paper) could derive from this mechanism of CpG enrichment at promoter level. It was found that CpG dinucleotides increase towards the TSS with the same rate of the other three dinucleotides, i.e. GpC, CpC and GpG, despite they are still relatively underexpressed. In particular, all the four dinucleotides provide comparable contributions to the increase in GC content close to the TSS (see Fig S11). We want to point out that this finding is coherent with the recent novel evolutionary model for the origin of CpG islands in promoter regions [79]. The absence of indications in favor of the selection on CpG densities suggests that the CpG increase at promoter level may be the result of the GC enrichment and not viceversa. This notwithstanding, the functional involvement of CpG is not excluded. In fact, the regulation through

methylation of CpG could be the indirect cause of CpG hypomethylation and slow decay in a large number of promoter regions [79].

Global statistics on regular sequences in *H. sapiens*

In this section we report the data about the total number of regular sequences found in the clusters of *H. sapiens* (Tab. S1) and their length distribution (Fig. S12). We observe an exponential decay in the length distribution, where long regular sequences are much less frequent.
